# The complete plastid genomes of *Ophrys iricolor* and *O*. *sphegodes* (Orchidaceae) and comparative analyses with other orchids

**DOI:** 10.1371/journal.pone.0204174

**Published:** 2018-09-18

**Authors:** Luca Roma, Salvatore Cozzolino, Philipp M. Schlüter, Giovanni Scopece, Donata Cafasso

**Affiliations:** 1 Department of Biology, University Federico II of Naples, Complesso Universitario Monte Sant’Angelo, Naples, Italy; 2 Department of Systematic and Evolutionary Botany, University of Zurich, Zollikerstrasse 107, Zurich, Switzerland; 3 Institute of Botany, University of Hohenheim, Garbenstraße 30, Stuttgart, Germany; University of Naples Federico II, ITALY

## Abstract

Sexually deceptive orchids of the genus *Ophrys* may rapidly evolve by adaptation to pollinators. However, understanding of the genetic basis of potential changes and patterns of relationships is hampered by a lack of genomic information. We report the complete plastid genome sequences of *Ophrys iricolor* and *O*. *sphegodes*, representing the two most species-rich lineages of the genus *Ophrys*. Both plastomes are circular DNA molecules (146754 bp for *O*. *sphegodes* and 150177 bp for *O*. *iricolor*) with the typical quadripartite structure of plastid genomes and within the average size of photosynthetic orchids. 213 Simple Sequence Repeats (SSRs) (31.5% polymorphic between *O*. *iricolor* and *O*. *sphegodes*) were identified, with homopolymers and dipolymers as the most common repeat types. SSRs were mainly located in intergenic regions but SSRs located in coding regions were also found, mainly in *ycf1* and *rpoC2* genes. The *Ophrys* plastome is predicted to encode 107 distinct genes, 17 of which are completely duplicated in the Inverted Repeat regions. 83 and 87 putative RNA editing sites were detected in 25 plastid genes of the two *Ophrys* species, all occurring in the first or second codon position. Comparing the rate of nonsynonymous (dN) and synonymous (dS) substitutions, 24 genes (including *rbc*L and *ycf*1) display signature consistent with positive selection. When compared with other members of the orchid family, the *Ophrys* plastome has a complete set of 11 functional *ndh* plastid genes, with the exception of *O*. *sphegodes* that has a truncated *ndh*F gene. Comparative analysis showed a large co-linearity with other related Orchidinae. However, in contrast to *O*. *iricolor* and other Orchidinae, *O*. *sphegodes* has a shift of the junction between the Inverted Repeat and Small Single Copy regions associated with the loss of the partial duplicated gene *ycf*1 and the truncation of the *ndh*F gene. Data on relative genomic coverage and validation by PCR indicate the presence, with a different ratio, of the two plastome types (i.e. with and without *ndh*F deletion) in both *Ophrys* species, with a predominance of the deleted type in *O*. *sphegodes*. A search for this deleted plastid region in *O*. *sphegodes* nuclear genome shows that the deleted region is inserted in a retrotransposon nuclear sequence. The present study provides useful genomic tools for studying conservation and patterns of relationships of this rapidly radiating orchid genus.

## Introduction

Plastids such as chloroplasts are important plant organelles involved in the photosynthetic process thus providing essential energy to plants [1]. Plastids have small circular genomes, ranging from 135 to 160 kb [2–4]. Most angiosperm plastid genomes so far annotated have a quadripartite structure containing two copies of Inverted Repeat (IR) regions, separating a Large Single Copy (LSC) and Small Single Copy (SSC) regions [5–7]. Recently, with the extraordinary advances in sequencing platforms, many plastid genomes have been annotated and have provided valuable tools for the understanding of plant phylogenies and genome evolution e.g. [8]. Plastid structure and gene order are generally stable, and the rate of nucleotide substitution is slow [9] so that plastid genomes were traditionally considered to have experienced rearrangements rarely enough to be suitable to demarcate major plant groups [10]. Nonetheless, several angiosperm lineages show extensive gene order changes in plastid genomes that are often correlated with increased rates of nucleotide substitutions and gene and/or multiple intron losses [11, 12]. These rearrangements in the plastid genome have been found to be often associated with repeated sequences [2].

The family Orchidaceae consists of more than 700 genera and approximately 28,000 species [13], which are distributed in a wide variety of habitats. So far, several complete plastid genomes have been annotated in different orchid lineages. These studies revealed that Orchidaceae often underwent accelerated plastome evolution including large inversions, shifts in boundaries between IRs and the two single copies, indels, intron losses, and pseudogene formation by stop codons often associated with shifts from heterotrophy to parasitism/heterotrophism [14,15]. Compared to other angiosperms, photosynthetic orchids were also found particularly variable in the conservation of *NADH* dehydrogenase *(ndh)* genes [16], that encode components of the thylakoid complex involved in the redox level of the cyclic photosynthetic electron transporters.

The number of intact and degraded *ndh* genes present in the orchids plastomes varies even among closely related species suggesting that this specific gene class may be actively degraded in Orchidaceae [17]. This is not surprising as gene transfer from plastid to nucleus is known to occur frequently during evolutionary processes as even the complete loss of some plastid-encoded *ndh* genes seems to not affect the plant life [15]. Indeed, there is no clear-cut evidence of phylogenetic signal in the pseudogenization or loss of the *ndh* genes. For instance, no correlation with phylogeny was found for *ndh* genes loss in the Epidendroideae lineages while related species of Oncidiinae show a consistent loss of two *ndh* genes (*ndh*F and *ndh*K) and pseudogenization by gene truncation of other five genes (*ndh*A, D, H, I and J) [18].

The IR/SC junctions represent another hotspot of orchid plastome evolution, with the rearrangement of flanking regions leading to expansion or contraction of the inverted repeat regions. Different types of junctions have been reported in orchids, with considerable variation particularly in the *ycf*1 gene [19]. It has been hypothesized that the exhibited usage bias of A/T base pairs typical of all known orchid *ycf*1 genes would render less stable the DNA in the *ycf*1 gene thus leading to the higher recombination of IR/SSC junction [20]. This often leads to a consequent partial or complete degradation of the *ndh*F gene, or even, in some case, to its transfer to mitochondrial DNA by intraorganellar recombination [17].

Despite Orchidaceae represents approximately 1/8 of all flowering plants [13], most published plastid sequences belong to tropical orchid lineages, while there is a remarkable dearth of information for the important temperate terrestrial subtribe Orchidinae with only two *Habenaria* and one *Platanthera* species plastomes having been annotated so far [17, 21]. With the aim to fill this gap, we sequenced the complete plastid genomes of *Ophrys iricolor* and *Ophrys sphegodes*. These species are representative of the two main diverging lineages of the Mediterranean *Ophrys*, a sexually deceptive genus belonging to the subtribe Orchidinae characterized by an elevated taxonomic complexity due to a very fast radiation by pollinator shifts [22, 23]. The specific aims of the present study were to (i) annotate the complete plastid genome sequences of two *Ophrys* species, (ii) evaluate the homology between these two plastomes, (iii) investigate any significant characteristics suggesting plastome rearrangement in *Ophrys* and their phylogenetic signal, and (iv) explore significant changes in gene content and gene order in the subtribe Orchidinae compared to other orchid subtribes.

## Materials and methods

### Genome sequencing, assembling and annotation

DNA was extracted from a specimen of *Ophrys iricolor* (collected between Miamou and Agios Kyrillos, Crete, Greece; N34.9693, E24.9154; under permit number 118565/3022 issued by the Ministry of Environment and Energy in Athens on 13.02.2015) and from a specimen of *Ophrys sphegodes* (collected between Cagnano Varano and San Nicandro Garganico, Apulia, Italy; N41.9133, E15.6784 under permit number 173 issued by the National Park of Gargano in Monte Sant’Angelo (FG) on 12.01.2016). Whole genomic libraries were sequenced in paired-end mode, 2 x 150 bp, using the Illumina HiSeq 4000 platform (Illumina Inc., San Diego, CA, USA) at the Functional Genomics Centre Zurich (Switzerland). The obtained reads were trimmed using the software TRIMMOMATIC v. 0.36 [24] and the resulting trimmed reads (309,012,252 reads for *O*. *sphegodes* and 251,959,572 reads for *O*. *iricolor*) were *de novo* assembled using NOVOPLASTY v. 2.5.2 [25]. The gene annotation of the *Ophrys* plastid genomes was carried out using the software GESEQ v. 1.42 [26] and BLAST v. 2.6.0 [27] searches. From this initial annotation analysis, putative starts and stops of the gene exons, along with the positions of the related introns, were determined based on comparisons to homologous genes in other plastid genomes [28]. All tRNA genes were verified by using tRNAscan-SE server v. 1.3.1 [29]. The physical maps of the plastid circular genomes were drawn using Organellar Genome DRAW (OGDRAW) v. 1.2.1 [30]. The complete plastome sequences of *Ophrys sphegodes* and *O*. *iricolor* were deposited in the Sequence Reads Archive (NCBI-SRA) database under the accession number SRP148126. BLAST v. 2.6.0 [27, 31] was used to check whether deleted part of the *ndh*F gene in the *O*. *sphegodes* plastid genome was translocated into the nuclear genome. Reads were realigned against the assembled scaffolds of *O*. *sphegodes* nuclear genome (unpublished) using BWA v. 0.7.16 and converted in BAM [32] format using SAMtools v. 1.5 [33]. Finally, a BLASTX search was performed to annotate the nuclear *O*. *sphegodes* scaffold1075174.

### Genome structure, deletions validation, and repeat sequences

The software MAFFT v. 7.205 [34] and the Perl script Nucleotide MUMmer (NUCmer) available in MUMmer 3.0 [35] were employed to compare the plastome structures between *O*. *sphegodes* and *O*. *iricolor*. To detect putative errors in the *de novo* assemblies, the trimmed reads were mapped to the assembled genomes using the aligner BWA [32], converted to BAM format using SAMtools [33] and finally visualized using the IGV genome browser v. 2.4 [36]. To validate the deletion *in silico*, BAM files were further analysed using the software BEDtools coverage v. 2.21.0 [37] which generated a table in BED format containing an interval “windows” with coverage information across the two *Ophrys* plastomes. The BED file format was in turn used to visualize the sequencing coverage in regions of interest using the software CNView v. 1.0 [38]. To experimentally validate the *ndh*F deletion in *O*. *sphegodes*/*O*. *iricolor*, we designed primers for both the flanking and internal regions of *ndh*F from the assembled plastomes (S1A Fig). With these primers, we PCR amplified DNAs of *O*. *sphegodes* and *O*. *iricolor* from different localities and of *O*. *incubacea* and *O*. *fusca*, as close relatives to *O*. *sphegodes* and *O*. *iricolor*, respectively and *O*. *insectifera* as distant related. PCR reaction conditions were as described in [39], with 5 ng of total DNA as template. Amplification products were visualized on 2% agarose gel using a 100 bp ladder as standard. PCR products and ladder were stained with ethidium bromide and photographed using a digital camera. Confirmatory sequences of the PCR products were done with ABI3130 automatic sequencer following manufacture instructions. Simple sequence repeats (SSRs) or microsatellites were detected using the MIcroSAtellite (MISA) Perl script v. 1.0 [40]. Thresholds were set at eight repeat units for mononucleotide SSRs, four repeat units for di- and trinucleotide SSRs, and three repeat units for tetra-, penta- and hexanucleotide SSRs as done in [41]. We also analysed tandem repeat sequences from the plastid genomes of *O*. *sphegodes* and *O*. *iricolor* and searched for forward, reverse and palindromic repeats by using REPuter [42]. We limited the maximum computed repeats and the minimal repeat size to 50 and 8, respectively and with a Hamming distance equal to 1.

### Prediction of RNA editing sites and identification of positive signatures in plastid protein-coding genes

Potential RNA editing sites in protein-coding genes of *Ophrys* plastome were predicted by the program PREPACT v. 2.0 [43] using the following 30 highly homologous reference genes from *Phalaenopsis aphrodite*: *acc*D, *atp*A, *atp*B, *atp*F, *atp*I, *ccs*A, *clp*P, *mat*K, *pet*B, *pet*D, *pet*G, *pet*L, *psa*B, *psa*I, *psb*B, *psb*E, *psb*F, *psb*L, *rpl*2, *rpl*20, *rpl*23, *rpo*A, *rpo*B, *rpo*C1, *rpo*C2, *rps*2, *rps*8, *rps*14, *rps*16, and *ycf*3.

In order to identify putative genes under positive selection, the 67 protein-coding genes present in sixteen Orchidaceae plastomes (*Ophrys iricolor*, AP018716 *O*. *sphegodes* AP018717, *Cattleya crispata* NC_026568.1, *Corallorhiza odontorhiza* KM390021.1, *Cymbidium aloifolium* NC_021429.1, *Cypripedium japonicum* KJ625630.1, *Goodyera procera* NC_029363.1, *Habenaria pantlingiana* NC_026775.1, *Masdevallia coccinea* NC_026541.1, *Phalaenopsis aphrodite* NC_017609.1, *Anoectochilus emeiensis* NC_033895.1, *Apostasia wallichii* NC_030722.1, *Dendrobium officinale* KX377961.1, *Phragmipedium longifolium* KM032625.1, *Platanthera japonica* MG925368.1, *Vanilla planifolia* KJ566306.1) were downloaded from Genbank. We analysed all coding gene regions, except *ndh* genes, due to their frequent loss across the entire set of orchids listed here.

In order to build a reference phylogenetic tree, all genes were aligned using MAFFT software v. 7.205 [44] and were concatenated using MESQUITE software v. 3.5 [45]. PARTITION FINDER software v. 2.1.0 [46] was used in order to search the best evolution model for each gene and a reference phylogenetic tree was built using RAxML software v. 8.2.10 using 1000 bootstrap replicates [47]. The positive signatures were analysed using SELECTON server v. 2.4 (http://selecton.tau.ac.il/index.html; [48], *Ophrys iricolor* was used as query sequence (i.e. the plastome type without *ndh*F deletion) and codon alignment was done using the software MAFFT v. 7.205 [44] implemented in SELECTON software. The phylogenetic tree was set as input in SELECTON analyses and branch lengths were automatically optimized from the software. The gene divergence was estimated by the sum of total branch lengths that link the operational taxonomical units to the common ancestor of Orchidaceae species sampled here as done in [28]. SELECTON software generated for each gene as output the number of putative sites under positive selection. In order to test whether positive selection is operating on a protein, a Likelihood Ratio Test for positive selection was performed with the comparison of M8 (allows positive selection) against M8a (null model). We consider in our analysis only sites where possible positive selection was inferred (lower bound > 1 and test with probability < 0.01). P-values were adjusted for multiple testing in R (R Core Team) using FDR method in the *p*.*adjust* function.

## Results and discussion

### Genome organization and features

**Fig 1:**
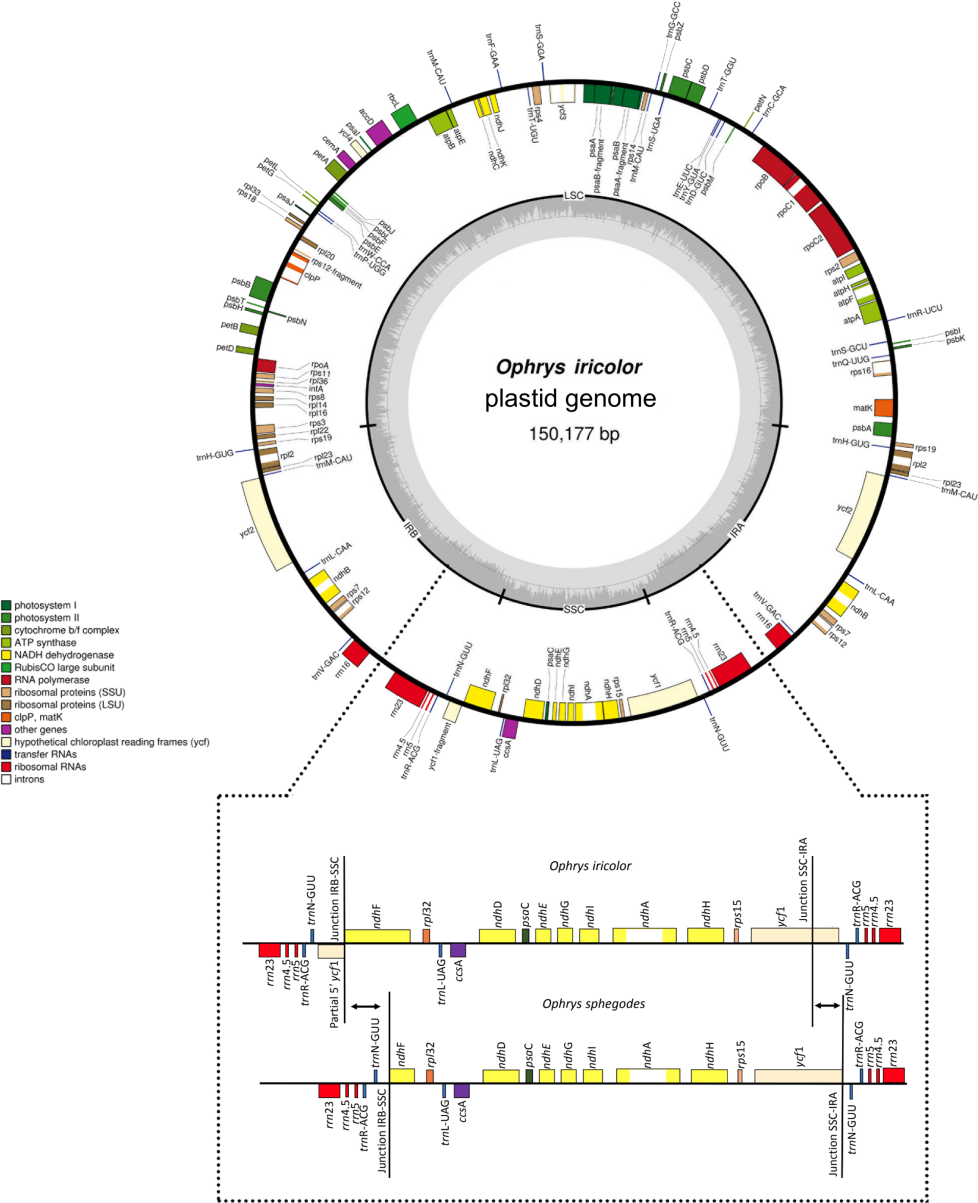
Gene map of *Ophrys sphegodes* (a) and *Ophrys iricolor* (b) plastid genomes. Genes drawn inside the circle are transcribed in the clockwise direction, and genes drawn outside are transcribed in the counter-clockwise direction. Different functional groups of genes are colour-coded. The darker grey in the inner circle corresponds to G/C content, and the lighter grey corresponds to A/T content. LSC, Large Single Copy; SSC, Small Single Copy; IRA/B, Inverted Repeat A/B. The enlargement shows that the loss of the partial duplicated gene of *ycf*1 and the truncation of *ndh*F gene in *O*. *sphegodes* are correlated with the shift of the junction between the IR and SSC.

The plastomes of the two *Ophrys* species are circular DNA molecules of 146,754 bp for *O*. *sphegodes* and 150,177 bp for *O*. *iricolor* with the typical quadripartite structure of plastid genomes of flowering plants (Fig 1): a pair of inverted repeats of 25,052 bp and 26,348 bp, respectively, separated by a large single copy (LSC) region (80,471 bp and 80,541, respectively) and a small single copy (SSC) region of 16,179 bp and 16,940 bp, respectively for *O*. *sphegodes* (DDBJ accession number AP018717) and *O*. *iricolor* (DDBJ accession number AP018716). The size of the *Ophrys* plastid genome was comparable to other published plastomes of photosynthetic orchids. The plastomes of the two *Ophrys* species are largely collinear with the exception of a large deletion in the *ndh*F gene in *O*. *sphegodes*.

The percentage of plastid reads in total WGS data was 5.43% for *O*. *sphegodes* and 1.96% for *O*. *iricolor*. The lowest average coverage of the assembled plastid genomes used was 13,673x for *O*. *sphegodes* and 3,816x for *O*. *iricolor*. The G/C contents were 37.14% and 36.4% respectively for *O*. *sphegodes* and *O*. *iricolor*, similar to other angiosperms (Table 1). The *Ophrys* plastome is predicted to encode 107 distinct genes, 17 of which are completely duplicated in the IR regions resulting in a total of 124 genes (Table 2). The annotation revealed distinct protein-coding genes (seven of them completely duplicated, namely *ndh*B, *rpl*2, *rpl*23, *rps*7, *rps*12, *rps*19 and *ycf*2), 30 distinct tRNAs genes (five of them duplicated, *trn*H-GUG, *trn*L-CAA, *trn*N-GUU, *trn*R-ACG, *trn*V-GAC and one triplicated *trn*M-CAU), and four distinct rRNA genes (all of them completely duplicated: *rrn*4.5, *rrn*5, *rrn*16 and *rrn*23). A truncated gene *ndh*F, was identified in *O*. *sphegodes* but not in *O*. *iricolor*. Ten genes contain one intron (*atp*F, *ndh*A, *ndh*B, *pet*B, *pet*D, *rpl*2, *rpl*16, *rps*12, *rps*16 and *rpo*C1) and two genes (*clp*P and *ycf*3) contain two introns.

**Table 1:** Comparison of two *Ophrys* plastid genomes.

	*Ophrys sphegodes*	*Ophrys iricolor*
Plastid reads	16,782,955 bp	4,952,605 bp
Average plastid coverage	13,673 x	3,816 x
G/C percentage	37.14%	36.4%
Large Single Copy Region	80,471 bp	80,541 bp
Small Single Copy Region	16,179 bp	16,940 bp
Inverted Repeats	25,052 bp	26,348 bp

**Table 2:** List of genes identified in the plastomes of *Ophrys iricolor* and *Ophrys sphegodes*.

Group of gene	Name of gene
Ribosomal RNA genes	*rrn*16^a^; *rrn*23^a^; *rrn*4.5^a^; *rrn*5^a^
Transfer RNA Genes	*trn*C-GCA; *trn*D-GUC; *trn*E-UUC; *trn*F-GAA; *trn*G-CCC; *trn*G-GCC; *trn*H-GUG^a^; *trn*L-CAA^a^; *trn*L-UAG; *trn*M-CAU^c^; *trn*N-GUU^a^; *trn*P-UGG; *trn*Q-UUG; *trn*R-ACG^a^; *trn*R-UCU; *trn*S-GCU; *trn*S-UGA; *trn*S-GGA; *trn*T-GGU; *trn*T-UGU; *trn*V-GAC^a^; *trn*W-CCA; *trn*Y-GUA
Small subunit of ribosome	*rps*2; *rps*3; *rps*4; *rps*7^a^; *rps*8; *rps*11; *rps*12^a^; *rps*14; *rps*15; *rps*16; *rps*18; *rps*19^a^
Large subunit of ribosome	*rpl*2^a^; *rpl*14; *rpl*16; *rpl*20; *rpl*22; *rpl*23^a^; *rpl*32; *rpl*33; *rpl*36
DNA-dependent RNA polymerase	*rpo*A; *rpo*B; *rpo*C1; *rpo*C2
*Genes for photosynthesis*:	
Subunits of photosystem I (PSI)	*psa*A; *psa*B; *psa*C; *psa*I; *psa*J; *ycf*3; *ycf*4
Subunits of photosystem II (PSII)	*psb*A; *psb*B; *psb*C; *psb*D; *psb*E; *psb*F; *psb*H; *psb*I; *psb*J; *psb*K; *psb*L; *psb*M; psbN, *psb*T; *psb*Z
Subunits of cytochrome b_6_f	*pet*A; *pet*B; *pet*D; *pet*G; *pet*L; *pet*N
Subunits of ATP synthase	*atp*A; *atp*B; *atp*E; *atp*F; *atp*H; *atp*I
Subunits of NADH dehydrogenase	*ndh*A; *ndh*B^a^; *ndh*C; *ndh*D; *ndh*E; *ndh*F^b^; *ndh*G; *ndh*H; *ndh*I; *ndh*K; *ndh*J
Large subunits of Rubisco	*rbc*L
*Other genes*:	
Maturase	*mat*K
Envelope membrane protein	*cem*A
Subunit of acetyl-CoA carboxylase	*acc*D
C-type cytochrome synthesis gene	*ccs*A
Protease	*clp*P
Component of TIC complex	*ycf*1^d^
Translation initiation factor IF-1	*inf*A
Genes of unknown function	*ycf*2^a^

^a^ Duplicated gene

^b^ Truncated in *O*. *sphegodes*

^c^ triplicated gene

^d^ partially duplicated in *O*. *iricolor*

### Repeat sequence detection

The occurrence, type, and distribution of SSRs in *Ophrys* plastomes were analysed. In total, 213 SSRs were identified in *O*. *sphegodes* and *O*. *iricolor*. Three of these microsatellites occurred in the sequence portion that is deleted in *O*. *sphegodes* plastome (S1 Table). Homopolymers and dipolymers were the most common SSRs with, respectively, 71% and 24% occurrence. Seven and nine SSRs were present in compound formation in *O*. *iricolor* and *O*. *sphegodes*, respectively. Furthermore, the majority of *O*. *sphegodes* and *O*. *iricolor* SSRs are located in IGS regions (56.2% and 55%), followed by coding sequences (38.2% and 38%) and introns (5.6% and 7%), respectively. SSRs located in coding regions were found mainly in *ycf1* and *rpoC2* genes. A comparison of SSRs found in the two *Ophrys* species showed that 67 SSRs (31.5% of the total) were polymorphic between the two species. Among these polymorphic SSRs, 46 were located in the IGS regions, 5 in introns and 16 in genes (S1 Table).

*Ophrys sphegodes* contains 15 directed repeats, 9 inverted repeats, 3 complementary repeats and 21 palindromic repeats, whose lengths range from 18 to 60 bp. *Ophrys iricolor* contains 15 directed repeats, 27 palindromic repeats, 2 complementary repeats and 5 inverted repeats, whose lengths range from 20 to 60 bp. Most of the *O*. *iricolor* and *O*. *sphegodes* repeats were located in IGS regions (65.3% and 66.7% respectively), others were located in genes (22.4%, in *ycf*2, *pet*G, *ndh*C, *psa*A and 22.9%; in *psb*I, *ndh*C, *ycf*2, *ndh*A respectively) and introns (12.3% and 10.4% in *clp*P and *rps*16 intron respectively).

### RNA editing sites prediction and positive signatures of adaptive evolution

The RNA editing is a post-transcriptional modification typical of plastid and mitochondrial DNA. The process originated early during the evolution of land plants and several RNA editing sites have been maintained or lost during angiosperms evolution [49, 50]. In our analysis, PREPACT found a total of 83 and 87 putative RNA editing sites in 25 genes in *O*. *sphegodes* and *O*. *iricolor* respectively (S2 Table), in line with previous report for other orchids [51]. The RNA editing sites predicted for plastid genes of *Ophrys sphegodes* and *Ophrys iricolor* occur in the first or second codon position with all nucleotide changes being from cytidine (C) to uridine (U), as very often reported in other angiosperms. In *O*. *sphegodes* the genes predicted to have RNA editing sites are *mat*K (12 sites), *rpo*C1 (9 sites), *rpo*C2 (8 sites), *rpo*B (8 sites), *acc*D (6 sites), *rpo*A (4 sites), *atp*A (4 sites), *rpl*2 (3 sites) *rpl*20 (3 sites), *atpI* (3 sites), *ccs*A (3 sites), *ycf*3 (3 sites), *clp*P (3 sites), *pet*B (2 sites), *rps*16 (2 sites) and the *atp*F, *pet*D, *pet*L, *psa*B, *psa*I, *psb*F, *rpl*23, *rps*2, *rps*8 and *rps*14 genes with only one site. In *O*. *iricolor* the genes predicted were the same as *O*. *sphegodes* with few differences: *ccs*A (5 sites), *atp*A (3 sites), *psa*B (2 sites), *rps*14 (2 sites), *atp*F (2 sites) (S2 Table) which suggest a general conservation of the RNA editing mechanism within *Ophrys* but also that RNA editing evolution accumulated enough differences to differentiate two *Ophrys* species. A previous study has also found that the number of RNA editing sites predicted for protein-coding genes in orchids species is high in comparison with other monocots [51].Likelihood ratio test between a null model and an alternative model carried out following [52] shows that 24 genes are under positive selection (S3 Table); overall, the most divergent genes have the stronger signatures of positive selection (S2 Fig). In details, the positively selected genes were involved in different essential functions such as photosynthesis, PSII (*psb*A, *psb*B, *psb*E, *psb*H, *psb*M, *psbN* genes), large subunits of rubisco (*rbc*L), ATP synthase (*atp*I gene), cytochrome b6f (*pet*B gene), subunits of RNA polymerase (*rpo*A, *rpo*B, *rpo*C1, *rpo*C2 genes), RNA maturation (*mat*K gene), ribosomal proteins (*rpl*20, *rpl*22, *rpl*32, *rpl*33, *rps*12, *rps*19 genes), fatty acid biosynthesis (*acc*D gene), cytochrome biosynthesis (*ccs*A gene), import of protein in the plastid (*ycf*1 gene), and unknown function (*ycf*2 gene). The high number of genes containing positive signatures (including the *rbc*L gene) among photosynthesis-related genes are consistent with previous observation on other monocots and may be related to the recent increase of diversification rate following adaptation to different ecological conditions. [53]. In particular, and as already suggested for other monocots as Arecaceae, many tropical orchid species grow as epiphytes in tropical forests and are shade adapted. The transition to the terrestrial habitus of all temperate orchid lineages (as *Ophrys*) may have promoted a new selective pressure for improving the photosynthesis efficiency under the new terrestrial ecological conditions [52].

Interestingly, some positively selected sites that were identified in our study (e.g., the *acc*D and *ycf*1genes) have been found very variable also in other orchids and flowering plants [54]. In particular *acc*D gene is a conserved plastid gene involved in de novo synthesis of fatty acids [55] and is essential for chloroplast functionality, leaf development and longevity [56]. Therefore *acc*D has been associated in a significant manner with adaptation to the environment, including factors such as temperature, light, humidity, and atmosphere [57].

On the other hand, *ycf*1 is one of the largest plastid genes and it has been found extremely divergent in orchids plastomes [19], likely because of its position at IR/SC junction that generates large variation in sequence length and pseudogenes [58] as also found in our study.

### Genomic comparison of *Ophrys* with other orchid plastomes

The *Ophrys* plastid genome is fully collinear both in gene order an gene orientation with the other available Orchidoideae. When compared with representative species belonging to the different subfamilies of the Orchidaceae (i.e., Epidendroideae, Cypripedioideae, Vanilloideae and Apostasioideae), we found that Cypripedioideae, Epidendroideae, Vanilloideae and Orchidoideae are largely collinear in plastid sequence with a few small exceptions: an inversion of the *psb*M—*pet*N gene order in Epidendroideae and a gene inversion in the SSC of *Vanilla* (S3 Fig).

In contrast to these four tribes, large rearrangements in gene order have been found in the supposed basal smaller tribe of Apostasioideae. However, under the assumption that the common plastid types observed in most orchids represent the primitive state, it is likely that the rearrangements found in *Apostasia wallichii* and *Apostasia odorata* (but not in the related *Neuwiedia* [59]) may be due to recent, terminal autoapomorphic changes rather than being representative of the ancestral gene order of the orchid family.

As many *ndh* genes had either truncations or indels, resulting in frameshifts or pseudogenes in several orchid plastomes, we also compared *ndh* genes in the different tribes. *Ophrys iricolor*, like other Orchidoideae, has the complete set of *ndh* plastid genes, i.e. 11 functional genes, which is different from *Apostasia wallichii* and *Vanilla planifolia* in which the *ndh*B gene is truncated and from *Vanilla planifolia* where all other 10 *ndh* subunits are deleted. The presence of *ndh* genes within terrestrial Orchidoideae is ubiquitous, which contrasts with the extensive variation in presence/absence of *ndh* genes found within tropical orchid genera (see *Cymbidium* [17]). The functional role of the *ndh* genes seems closely related to the land adaptation of photosynthesis so they have been conserved in terrestrial, temperate orchid plastomes whereas they are partially lost in epiphytic, tropical orchid plastomes [60].

### Boundaries between single copy and inverted repeat regions

**Fig 2:**
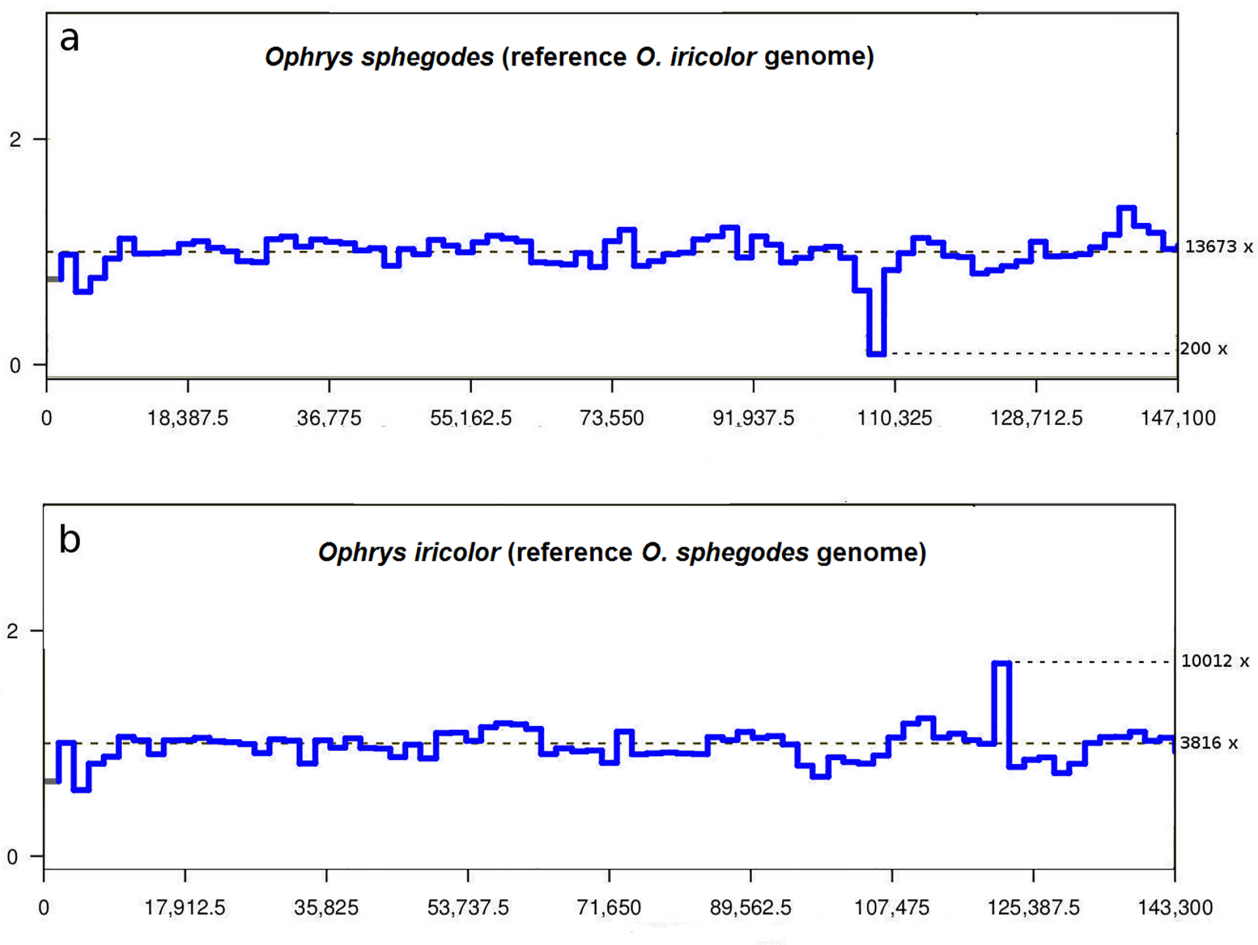
*In silico* validation of *ndh*F deletion (using software CNView) comparing *O*. *sphegodes* plastid reads against reference genome of *O*. *iricolor* (a) and *O*. *iricolor* plastid reads against reference genome of *O*. *sphegodes* (b). Y-axis represents normalized coverage values.

Expansion or contraction of the IR region is one of the main causes of size variation among angiosperm plastid genomes [61] and it has found to be variable even among related orchid species as, for instance, within the *Cymbidium* genus [17]. The multiple genome alignment analysis using plastome sequences of *O*. *sphegodes* and *O*. *iricolor* revealed the loss of a *ycf*1 fragment in the IR and partial deletion of the *ndh*F gene in *O*. *sphegodes* (S4 Fig). *In silico* validation confirmed the partial *ndh*F gene loss in *O*. *sphegodes* and demonstrated that part of the *ycf*1 gene is duplicated in *O*. *iricolor*, which does not occur in *O*. *sphegodes* (Fig 2).

In *O*. *sphegodes*, the loss of the partial duplicated gene of *ycf*1 and the partial deletion of *ndh*F gene are correlated with the shift of the junction between the IR and SSC (Fig 1) with a pattern very similar to some *Cymbidium* species [17]. High sequence variability, especially in the *ycf*1 gene at IR-SSC junction, have been frequently observed as a result of expansion and contractions events by gene conversion [62, 63]. While *in silico* validation by CNVIEW largely confirms the occurrence of the *ndh*F deletion in *O*. *sphegodes*, however, approximately 2% of *O*. *sphegodes* reads map on the plastid region corresponding to *O*. *iricolor* plastome type (i.e. where complete *ndh*F occurs). At the same time, IGV also reveals that 888 of *O*. *iricolor* reads map on the junction with *ndh*F deletion (i.e. corresponding to *O*. *sphegodes* plastome type). Thus, to confirm the occurrence of *ndh*F deletion in *O*. *sphegodes*/*O*. *iricolor*, we amplified DNA with primers for both the flanking and internal regions of *ndh*F. Further, to rule out any possible cross contamination (during the NGS steps) as cause of presence of both plastome types in both *Ophrys* species, different accessions were used in PCR validation. PCR amplifications with primers flanking *ndh*F yielded two amplicons in *O*. *iricolor*: a small one (0.25 Kb), corresponding to the plastid fragment with the *ndh*F deletion, and a larger amplicon (3.25 Kb) containing the undeleted *ndh*F gene. Only the small plastid fragment with the *ndh*F deletion (primers F1/R1) was detected in *O*. *sphegodes*. To exclude, in *O*. *sphegodes*, that the small fragment was selectively amplified due to its shorter size and higher copy number, we also amplified *O*. *sphegodes* and *O*. *iricolor* (as control) with primers located within the *ndh*F deletion (primers F2/R2). Contrary to expectation (i.e. no amplification in *O*. *sphegodes*) both species successfully amplified a 1.2 Kb fragment. However, the two species differed in their amplicon yield, i.e. we obtained a stronger amplification band in *O*. *iricolor* compared to *O*. *sphegodes* (S1 Fig b). Taken together, this suggest that both species contained copies with and without the *ndh*F deletion but with a different relative representation (high proportion of deletions in *O*. *sphegodes* and low in *O*. *iricolor*). The fact that all examined members of *O*. *sphegodes* and *O*. *iricolor* lineages (including the basal *O*. *insectifera*) share a similar PCR amplification pattern suggests that the deletion of *ndh*F has likely occurred only once during the early evolution of the genus *Ophrys*, i.e. immediately before the separation of the two main lineages. The presence of two plastome types (with a different relative representation) across the two lineages represents an unusual case of maintenance of plastid heteroplasmy likely established as consequence of retention of ancestral polymorphism or of plastid capture by hybridization. Both processes have been commonly suggested to explain the unusual genomic admixture detected among *Ophrys* species as they are characterized by very rapid radiation and recurrent hybridization [64, 65].

### Genomic localization of deleted *ndh*F gene in *O*. *sphegodes* nuclear genome

**Fig 3:**
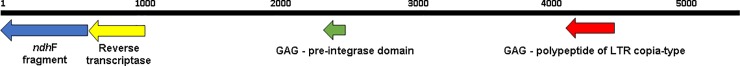
Results of BLASTX search of scaffold1075174 (length 5,436 bp): Putative domain hits are indicated by the colored arrows.

BLAST search of the assembly for the deleted *ndh*F region from the plastid genome of *O*. *sphegodes* found the nuclear scaffold1075174 (length 5,436 bp) with a score of 924 and e-value of 0.0. Reads of whole genome sequencing were mapped against scaffold1075174 to check whether some reads overlap with the junction between plastid deleted region and the remaining part of this scaffold. A total of 124,961 reads mapped on the scaffold. BLASTX search for the scaffold1075174 (after excluding the deleted plastid region) revealed the presence of a reverse transcriptase, a GAG pre-integrase domain, and the gag-polypeptide of LTR copia-type. Twelve reads map on the junction between *ndh*F and the reverse transcriptase so confirming the connection between the two parts. This result represents a clear indication that the deleted plastid region has been inserted in a retrotransposon nuclear sequence of *O*. *sphegodes* (Fig 3). Most of the repetitive DNA in available orchid genomes are gypsy- and copia-like retrotransposons [66] and their activity is likely to significantly contributed to the orchid large genome size [67].

## Conclusions

The complete plastid genomes provided here for two taxa from the rapidly evolving orchid genus *Ophrys* represents a source of novel information that can help resolve evolutionary questions. While the plastid gene order and organization reveal the signal of phylogenetic relationships among main species groups in this genus, the highly variable SSRs and tandem repeats with suitable level of intraspecific variation can be used as markers in phylogeographic and speciation studies among those closely related species. These relationships can now be explored with the novel genomic resources available today.

## Supporting information

S1 Table Distribution of simple sequence repeat (SSR) in *Ophrys sphegodes* and *O. iricolor* plastid genomes. IGS: intergenic spacer.
(DOCX)

S2 Table List of RNA editing sites predicted in protein-coding genes of *Ophrys* plastomes using PREPACT program. High dashes indicate absence of RNA editing, * stop codon.
(DOCX)

S3 Table Positive selection sites identified with selecton with d.f. = 1. “Null” and “positive” columns list likelihood values obtained under the models M8a (null model) and M8 (positive selection), respectively.
(DOCX)

S1 Fig PCR validation of the *ndh*F deletion. PCR amplifications using (a) F1 and R1 primers; (b) F2 and R2 primers. M = marker II (λ DNA / *Hind* III digested); 1 = *O*. *fusca* Campania, 2 = *O*. *fusca* Tuscany, 3 = *O*. *iricolor* Greece; 4 = *O*. *sphegodes* Campania; 5 = *O*. *sphegodes* Apulia; 6 = *O*. *incubacea* Apulia; 7 = *O*. *insectifera* Spain. A dotted line represents the IRB-SSC junction. Primer sequences:
F1: 5’—GCTCCGTTCCATGCCTCATT– 3’
R1: 5’–TCGTCGTATGTGGGCTTTCC– 3’
F2: 5’–TTAGCAATTGCACCGACAAA– 3’
R2: 5’–TCTGTTTCCACCGGACAG– 3’.
(TIFF)

S2 Fig Molecular evolution analyses of *Ophrys* plastid genes: a) divergence of protein-coding genes (gene divergence was estimated by the sum of total branch lengths in each gene tree inferred, mean ± SD); b) number of putative sites under positive selection.
(TIF)

S3 Fig Comparison of gene rearrangements in the plastid genomes among 10 species representative of the five Orchid subfamilies. Genes are indicated in the colored boxes. Boxes colors represent gene families: purple = photosystem I; yellow = photosystem II; orange = NADH-dehydrogenase; light green = ribosome large subunit; light blue = ribosome small subunit; light red = rubisco subunit; red = RNA polymerase; green = ATP synthase; pink = cytochrome b/f complex; dark grey = acetyl-CoA carboxylase; light grey = hypothetical plastid reading frame (ycf series), protease, translation initiation factor IF-1; dark purple = maturase; dark blue = envelope membrane protein.
(TIFF)

S4 Fig Dot-plot analyses of *Ophrys sphegodes* and *O. iricolor* plastid genomes using Mummer software. A positive slope indicates that compared sequences are in the same orientation; a negative slope indicates that compared sequences can be aligned, but their orientation is opposite. Red: Sequences in the same direction; Blue: inversions.
(JPG)
