# Supplementary material for: The complete plastid genomes of Ophrys iricolor and O. sphegodes (Orchidaceae) and comparative analyses with other orchids
Source: PLoS One. 2018 Sep 18;13(9):e0204174. doi: 10.1371/journal.pone.0204174 (PMC6143245; doi:10.1371/journal.pone.0204174)
Supplement: S1 Table — IGS: intergenic spacer. (DOCX) [file pone.0204174.s001.docx]

S1 Table Distribution of simple sequence repeat (SSR) in *Ophrys sphegodes* and *O. iricolor* plastid genomes. IGS: intergenic spacer.

| **SSR**  **nr.** | **Gene** | **SSR**  ***O. sphegodes*** | **start-end**  ***O. sphegodes*** | **SSR**  ***O. iricolor*** | **start-end**  ***O. iricolor*** |
| --- | --- | --- | --- | --- | --- |
| 1 | IGS *psb*A - *mat*K | (A)11 | 1294-1304 | (A)10 | 1294-1303 |
| 2 | IGS *psb*A - *mat*K | (T)11A(T)10 | 1317-1338 |  |  |
| 3 | IGS *psb*A - *mat*K |  |  | (T)14 | 1317-1330 |
| 4 | IGS *psb*A - *mat*K | (T)10 | 1600-1609 | (T)17 | 1582-1598 |
| 5 | *mat*K | (AT)4 | 2473-2480 | (AT)4 | 2462-2469 |
| 6 | *mat*K | (T)9 | 2782-2790 | (T)9 | 2771-2779 |
| 7 | *mat*K | (A)9 | 2935-2943 | (A)9 | 2924-2932 |
| 8 | IGS *mat*K - *rps*16 | (T)10 | 3352-3361 | (T)11 | 3349-3359 |
| 9 | IGS *mat*K - *rps*16 | (A)10 | 3676-3685 | (A)11 | 3683-3693 |
| 10 | IGS *mat*K - *rps*16 | (A)13 | 3890-3902 |  |  |
| 11 | IGS *mat*K - *rps*16 | (T)11 | 3967-3977 | (T)14 | 3975-3988 |
| 12 | IGS *mat*K - *rps*16 | (T)8 | 3987-3994 | (T)8 | 3998-4005 |
| 13 | IGS *mat*K - *rps*16 | (A)10(AAT)4 | 4163-4182 | (AAT)6 | 4179-4196 |
| 14 | IGS *mat*K - *rps*16 | (A)12 | 4246-4257 | (A)9 | 4260-4268 |
| 15 | IGS *mat*K - *rps*16 | (T)12 | 4287-4298 | (T)10 | 4295-4304 |
| 16 | IGS *mat*K - *rps*16 | (G)12 | 4583-4594 |  |  |
| 17 | IGS *mat*K - *rps*16 |  |  | (C)10(T)8 | 4574-4591 |
| 18 | IGS *mat*K - *rps*16 | (A)9 | 4614-4622 | (A)9 | 4623-4631 |
| 19 | IGS *mat*K - *rps*16 | (T)10 | 4715-4724 |  |  |
| 20 | *rps*16 |  |  | (A)11 | 5187-5197 |
| 21 | *rps*16 | (T)8 | 5245-5252 | (T)10 | 5254-5263 |
| 22 | *rps*16 | (TA)6 | 5546-5557 | (TA)5 | 5558-5567 |
| 23 | IGS *rps*16 - *trn*Q-UUG | (T)11 | 6052-6062 | (T)10 | 6062-6071 |
| 24 | IGS *rps*16 - *trn*Q-UUG | (A)9 | 6072-6080 | (A)9 | 6081-6089 |
| 25 | IGS *trn*Q-UUG - *psb*K | (A)10 | 6497-6506 | (A)11 | 6506-6516 |
| 26 | IGS *trn*Q-UUG - *psb*K | (A)18 | 6595-6612 | (A)13 | 6605-6617 |
| 27 | *psb*K | (T)9 | 6756-6764 | (T)9 | 6761-6769 |
| 28 | IGS *psb*K - *psb*I | (AT)4 | 6814-6821 | (AT)4 | 6819-6826 |
| 29 | IGS *psb*K - *psb*I | (GT)4 | 6933-6940 | (GT)4 | 6940-6947 |
| 30 | IGS *psb*K - *psb*I | (A)13 | 6965-6977 | (A)13 | 6972-6984 |
| 31 | *trn*S-GCU | (GA)4 | 7243-7250 | (GA)4 | 7250-7257 |
| 32 | IGS *trn*S-GCU - *trn*R-UCU | (TA)6 | 7596-7607 | (TA)6 | 7596-7607 |
| 33 | IGS *trn*S-GCU - *trn*R-UCU | (T)12 | 7738-7749 | (T)11 | 7742-7752 |
| 34 | IGS *trn*S-GCU - *trn*R-UCU | (T)9 | 7948-7956 | (T)11 | 7951-7961 |
| 35 | IGS *trn*S-GCU - *trn*R-UCU | (A)8 | 8074-8081 | (A)10 | 8079-8088 |
| 36 | IGS *trn*S-GCU - *trn*R-UCU | (T)8 | 8219-8226 | (T)8 | 8226-8233 |
| 37 | *atp*A | (GTCT)3 | 9969-9980 | (GTCT)3 | 9969-9980 |
| 38 | *atp*F | (T)8 | 11330-11337 | (T)10 | 11330-11339 |
| 39 | *atp*F | (A)9 | 11395-11403 | (A)8 | 11396-11403 |
| 40 | *atp*F | (A)8 | 11832-11839 | (A)8 | 11833-11840 |
| 41 | IGS *atp*H - *atp*I | (A)8 | 12331-12338 | (A)8 | 12332-12339 |
| 42 | IGS *atp*H - *atp*I | (A)10 | 12453-12462 | (A)9 | 12454-12462 |
| 43 | IGS *atp*H - *atp*I |  |  | (AT)4 | 12683-12690 |
| 44 | IGS *rps*2 - *rpo*C2 |  |  | (T)11 | 14517-14527 |
| 45 | IGS *rps*2 - *rpo*C2 | (A)10 | 14662-14671 | (A)10 | 14686-14695 |
| 46 | *rpo*C2 | (T)10 | 16545-16554 | (T)10 | 16559-16568 |
| 47 | *rpo*C2 | (T)10 | 16612-16621 | (T)10 | 16626-16635 |
| 48 | *rpo*C2 | (T)11 | 16718-16728 | (T)11 | 16732-16742 |
| 49 | *rpo*C2 | (A)9 | 16866-16874 | (A)9 | 16880-16888 |
| 50 | *rpo*C2 | (AT)4 | 18016-18023 | (AT)4 | 18030-18037 |
| 51 | *rpo*C2 | (AT)5 | 18106-18115 | (AT)5 | 18120-18129 |
| 52 | *rpo*C1 | (A)8 | 20568-20575 | (A)8 | 20582-20589 |
| 53 | *rpo*C1 | (T)8 | 21761-21768 | (T)8 | 21775-21782 |
| 54 | *rpo*B | (T)8 | 24435-24442 | (T)8 | 24449-24456 |
| 55 | *rpo*B | (T)9 | 25495-25503 | (T)10 | 25501-25510 |
| 56 | IGS *rpo*B - *trn*C-GCA | (A)13 | 26090-26102 | (A)10 | 26146-26155 |
| 57 | IGS *pet*N - *psb*M | (A)14 | 27078-27091 | (A)11 | 27131-27141 |
| 58 | IGS *pet*N - *psb*M | (TA)4 | 27130-27137 | (TA)4 | 27180-27187 |
| 59 | IGS *pet*N - *psb*M | (AT)4 | 27239-27246 | (AT)4 | 27289-27296 |
| 60 | IGS *pet*N - *psb*M | (TA)5 | 27403-27412 | (AT)6 | 27399-27410 |
| 61 | IGS *pet*N - *psb*M |  |  | (TA)5 | 27461-27470 |
| 62 | IGS *psb*M - *trn*D-GUC |  |  | (T)11 | 28192-28202 |
| 63 | IGS *psb*M - *trn*D-GUC | (T)9(TTTA)3* | 28126-28143 |  |  |
| 64 | IGS *trn*E-UUC – *trn*T-GGU | (TA)4 | 29157-29164 | (TA)4 | 29215-29222 |
| 65 | IGS *trn*E-UUC – *trn*T-GGU | (A)10 | 29477-29486 | (A)12 | 29531-29542 |
| 66 | IGS *trn*E-UUC – *trn*T-GGU | (AT)5 | 29736-29745 | (AT)5 | 29778-29787 |
| 67 | IGS *trn*T-GGU - *psb*D | (A)8 | 29939-29946 | (A)8 | 29981-29988 |
| 68 | IGS *trn*T-GGU – *psb*D | (TAAA)3 | 30195-30206 | (TAAA)3 | 30237-30248 |
| 69 | IGS *psb*B - *trn*S-UGA | (TA)4(T)9 | 33202-33218 | (TA)10(T)10 | 33211-33240 |
| 70 | IGS *psb*Z - *trn*G-GCC | (AT)4 | 33944-33951 | (AT)4 | 33966-33973 |
| 71 | IGS *trn*G-GCC - *trn*M-CAU | (AT)4 | 34234-34241 | (AT)4 | 34256-34263 |
| 72 | IGS *trn*G-GCC - *trn*M-CAU | (A)9 | 34270-34278 | (A)10 | 34292-34301 |
| 73 | IGS trnG-GCC - *trn*M-CAU |  |  | (A)9 | 34322-34330 |
| 74 | IGS *trn*M-CAU - *rps1*4 | (AT)4 | 34449-34456 | (AT)4 | 34474-34481 |
| 75 | IGS *rps*14 - *psa*B | (A)12 | 34976-34987 | (A)10 | 35001-35010 |
| 76 | IGS *psb*30 - *ycf*3 | (A)8 | 39724-39731 | (A)8 | 39747-39754 |
| 77 | IGS *psb*30 - *ycf*3 | (T)11 | 39869-39879 | (T)10 | 40596-40605 |
| 78 | *ycf*3 | (T)9 | 40571-40579 | (T)12 | 41894-41905 |
| 79 | *ycf*3 | (T)11 | 41868-41878 |  |  |
| 80 | *trn*S-GGA | (CT)4 | 42468-42475 | (CT)4 | 42495-42502 |
| 81 | IGS *rps*4 - *trn*T-UGU | (TA)4 | 43494-43501 | (TA)4 | 43521-43528 |
| 82 | IGS *trn*T-UGU - *trn*F-GAA | (AT)4G(A)9 | 43912-43929 | (AT)4G(A)10 | 43912-43930 |
| 83 | IGS *trn*T-UGU - *trn*F-GAA | (T)13 | 44169-44181 | (T)10 | 44060-44069 |
| 84 | IGS *trn*T-UGU - *trn*F-GAA | (A)12 | 44235-44246 | (A)16 | 44307-44322 |
| 85 | IGS *trn*T-UGU - *trn*F-GAA | (T)13AT(A)13 | 44679-44706 | (T)10AT(A)13 | 44748-44772 |
| 86 | IGS *trn*T-UGU - *trn*F-GAA | (A)10 | 44802-44811 | (A)9 | 44866-44874 |
| 87 | IGS trnT-UGU - *trn*F-GAA |  |  | (TTATA)3 | 45073-45087 |
| 88 | IGS *trn*T-UGU - *trn*F- GAA | (AG)4 | 45173-45180 | (AG)4 | 45234-45241 |
| 89 | IGS *trn*F-GAA - *ndh*J | (AT)4 | 45638-45645 | (AT)4 | 45692-45699 |
| 90 | IGS *trn*F-GAA - *ndh*J | (T)10 | 45873-45882 | (T)12 | 45923-45934 |
| 91 | IGS *ndh*C - *trn*M-CAU | (A)11 | 48357-48367 | (A)11 | 48416-48426 |
| 92 | IGS *ndh*C - *trn*M-CAU | (T)10 | 48510-48519 | (T)13 | 48569-48581 |
| 93 | IGS *ndh*C - *trn*M-CAU | (TA)4 | 48549-48556 | (TA)4 | 48606-48613 |
| 94 | IGS *atp*B - *rbc*L | (T)9 | 51739-51747 | (T)9 | 51787-51795 |
| 95 | IGS *atp*B - *rbc*L |  |  | (T)8 | 51934-51941 |
| 96 | IGS *atp*B - *rbc*L | (AT)4 | 51983-51990 | (AT)4 | 52096-52103 |
| 97 | IGS *rbc*L - *acc*D | (T)10 | 53711-53720 | (T)9 | 53816-53824 |
| 98 | IGS *rbc*L - *acc*D | (T)8 | 54150-54157 | (T)10 | 54254-54263 |
| 99 | IGS *rbc*L - *acc*D | (A)11 | 54265-54275 | (A)10 | 54371-54380 |
| 100 | *acc*D | (T)9(TAA)4* | 54708-54727 | (T)9(TAA)4* | 54813-54832 |
| 101 | *acc*D | (TG)4 | 55397-55404 | (TG)4 | 55502-55509 |
| 102 | IGS *acc*D - *psa*I | (CT)4 | 56003-56010 | (CT)4 | 56109-56116 |
| 103 | IGS *acc*D - *psa*I | (A)11 | 56020-56030 |  |  |
| 104 | IGS *acc*D - *psa*I | (TA)4 | 56207-56214 | (TA)4 | 56310-56317 |
| 105 | IGS *psa*I - *ycf*4 | (AT)4 | 56508-56515 |  |  |
| 106 | *ycf*4 | (T)8 | 56906-56913 | (T)8 | 56926-56933 |
| 107 | *cem*A | (A)8 | 57866-57873 | (A)8 | 57879-57886 |
| 108 | *cem*A | (AATG)3 | 58531-58542 | (AATG)3 | 58544-58555 |
| 109 | *pet*A | (C)8 | 59122-59129 | (C)8 | 59135-59142 |
| 110 | IGS *psb*E - *pet*L | (T)8 | 61622-61629 | (T)8 | 61631-61638 |
| 111 | IGS *psb*E - *pet*L | (AT)4 | 61732-61739 | (AT)4 | 61741-61748 |
| 112 | IGS *pet*G - *trn*W-CCA | (T)8 | 62184-62191 | (T)8 | 62193-62200 |
| 113 | IGS *pet*G - *trn*W-CCA |  |  | (A)8 | 62229-62236 |
| 114 | IGS *trn*P - UGG - *psa*J | (TA)5 | 62662-62671 | (TA)5 | 62671-62680 |
| 115 | IGS *psa*J - *rpl*33 | (T)10 | 63160-63169 | (T)9 | 63169-63177 |
| 116 | IGS psaJ - *rpl*33 |  |  | (T)11 | 63544-63554 |
| 117 | IGS *psa*J - *rpl*33 | (A)8 | 63330-63337 | (A)12 | 63593-63604 |
| 118 | IGS *psa*J - *rpl*33 | (T)12 | 63520-63531 |  |  |
| 119 | IGS *psa*J - *rpl*33 | (A)10 | 63547-63556 |  |  |
| 120 | IGS *rpl*33 - *rps*18 | (AT)4 | 63856-63863 | (AT)4 | 63904-63911 |
| 121 | IGS *rpl*33 - *rps*18 | (A)8 | 63905-63912 | (A)10 | 63953-63962 |
| 122 | IGS *rpl*33 - *rps*18 | (TA)4 | 63926-63933 |  |  |
| 123 | IGS *rpl*33 - *rps*18 |  |  | (TA)4TTT(TA)4 | 63976-63994 |
| 124 | *rps*18 | (AAAT)3 | 64245-64256 | (AAAT)3 | 64306-64317 |
| 125 | *rpl*20 | (T)8 | 64836-64843 | (T)8 | 64897-64904 |
| 126 | IGS *rpl*20 - *clp*P | (T)14 | 65588-65601 | (T)12 | 65649-65660 |
| 127 | (intron) *clp*P | (A)12 | 66224-66235 | (A)10 | 66283-66292 |
| 128 | (intron) *clp*P | (A)9 | 66394-66402 | (A)10 | 66451-66460 |
| 129 | (intron) *clp*P | (A)10 | 67175-67184 | (A)9 | 67233-67241 |
| 130 | (intron) *clp*P | (T)15 | 67212-67226 | (T)11 | 67269-67279 |
| 131 | (intron) *clp*P | (A)9 | 67266-67274 | (A)9 | 67322-67330 |
| 132 | (intron) *clp*P | (T)10 | 67461-67470 | (T)10 | 67517-67526 |
| 133 | (intron) *clp*P | (T)8 | 67719-67726 | (T)10 | 67781-67790 |
| 134 | IGS *clp*P - *psb*B | (AT)4 | 68210-68217 | (AT)4 | 68274-68281 |
| 135 | IGS *clp*P - *psb*B | (TA)4 | 68293-68300 | (TA)4 | 68365-68372 |
| 136 | *psb*B | (T)8 | 69336-69343 | (T)8 | 69408-69415 |
| 137 | IGS *psb*C - *psb*T | (TA)4 | 70405-70412 | (TA)4 | 70477-70484 |
| 138 | IGS *psb*C - *psb*T | (T)8 | 70589-70596 | (T)8 | 70661-70668 |
| 139 | *psb*N | (T)10 | 70832-70841 | (T)9 | 70903-70911 |
| 140 | IGS *psb*H - *pet*B | (A)8 | 71527-71534 | (A)8 | 71265-71272 |
| 141 | IGS *psb*H - *pet*B | (A)10 | 71759-71768 | (A)8 | 71597-71604 |
| 142 | (Intron) *pet*B |  |  | (A)11 | 71828-71838 |
| 143 | IGS *pet*B - *pet*D | (AT)4 | 72716-72723 | (AT)4 | 72786-72793 |
| 144 | (intron) *pet*D | (T)9 | 73144-73152 | (T)8 | 73214-73221 |
| 145 | IGS *pet*D - *rpo*A | (A)12 | 74338-74349 |  |  |
| 146 | *rps*11 | (T)8 | 75468-75475 | (T)9 | 76059-76067 |
| 147 | *rps*8 | (T)8 | 76766-76773 | (T)11 | 76247-76257 |
| 148 | IGS *rps*8 - *rpl*14 | (T)11 | 77038-77048 | (T)8 | 76828-76835 |
| 149 | IGS *rps*8 - *rpl*14 | (T)11 | 77055-77065 | (T)8 | 76878-76885 |
| 150 | IGS *rps*8 - *rpl*14 | (T)10 | 77651-77660 | (T)10 | 77117-77126 |
| 151 | *rpl*14 | (T)9 | 78241-78249 | (T)10 | 77722-77731 |
| 152 | (intron) rpl16 |  |  | (T)8 | 78312-78319 |
| 153 | (Intron) *rpl*16 | (TA)4 | 78279-78286 | (TA)4 | 78349-78356 |
| 154 | (Intron) *rpl*16 | (T)9 | 78807-78815 | (T)10 | 78877-78886 |
| 155 | (intron) *rpl*16 | (T)9 | 79016-79024 | (T)10 | 79087-79096 |
| 156 | (intron) *rpl*16 | (T)10 | 79179-79188 | (T)9 | 79258-79266 |
| 157 | IGS *rpl*16 - *rps*3 | (T)9AT(A)12 | 79317-79339 | (T)10AT(A)10 | 79395-79416 |
| 158 | *rps*3 | (TA)4 | 79829-79836 | (TA)4 | 79899-79906 |
| 159 | *rps*3 | (T)10 | 79911-79920 | (T)10 | 79981-79990 |
| 160 | IGS *rps*3 - *rpl*22 | (T)9 | 80072-80080 | (T)9 | 80142-80150 |
| 161 | *rpl*22 | (T)8 | 80186-80193 | (T)8 | 80256-80263 |
| 162 | IGS *rpl*22 - *rps*19 | (AAAAT)3 | 80607-80621 |  |  |
| 163 | *rps*19 | (T)9 | 80961-80969 | (T)9 | 81043-81051 |
| 164 | IGS *rps*19 - *rpl*2 | (T)11 | 80997-81007 | (T)11 | 81079-81089 |
| 165 | *ycf*2 | (GA)4 | 84329-84336 | (GA)4 | 84402-84409 |
| 166 | *ycf*2 | (A)8 | 85210-85217 | (A)8 | 85283-85290 |
| 167 | *ycf*2 | (A)9 | 86451-86459 | (A)9 | 86524-86532 |
| 168 | *ycf*2 | (GA)5 | 86472-86481 | (GA)5 | 86545-86554 |
| 169 | IGS *trn*L-CAA - *ndh*B | (TA)5 | 91320-91329 | (TA)5 | 91449-91458 |
| 170 | *ndh*B | (AG)4 | 92003-92010 | (AG)4 | 92182-92189 |
| 171 | IGS *rps*12 - *trn*V-GAC | (T)12 | 96643-96654 | (T)11 | 96860-96870 |
| 172 | IGS *rps*12 - *trn*V-GAC | (A)8 | 96694-96701 | (A)8 | 96910-96917 |
| 173 | *rrn*23 | (CT)4 | 102777-102784 | (CT)4 | 103001-103008 |
| 174 | IGS *trn*N - GUU - *ndh*F | (A)12 | 105847-105858 |  |  |
| 175 | IGS *trn*N - GUU - *ndh*F | (A)11 | 105959-105969 |  |  |
| 176 | *IGS trn*N-GUU - *ndhF* | (T)8 | 106334-106341 |  |  |
| 177 | *ndh*F |  |  | (A)10 | 107254-107263 |
| 178 | *ndh*F |  |  | (T)8 | 107622-107629 |
| 179 | *ndh*F |  |  | (A)9 | 107843-107851 |
| 180 | IGS *ndh*F - *rpl*32 |  |  | (T)12 | 109056-109067 |
| 181 | *rpl*32 | (T)9 | 106421-106429 |  |  |
| 182 | IGS *ndh*F - *rpl*32 |  |  | (A)9 | 109108-109116 |
| 183 | IGS *ndh*F - *rpl*32 |  |  | (A)9 | 109218-109226 |
| 184 | IGS *rpl*32 - *trn*L-UAG | (A)10 | 106551-106560 |  |  |
| 185 | IGS *rpl*32 - *trn*L-UAG | (A)11 | 106574-106584 | (A)11 | 109778-109788 |
| 186 | *rpl*32 |  |  | (T)9 | 109657-109665 |
| 187 | IGS *rpl*32 - *trn*L-UAG |  |  | (A)8 | 109828-109835 |
| 188 | IGS *trn*L-UAG - *ccs*A | (AT)4 | 106944-106951 |  |  |
| 189 | *ccs*A | (T)9 | 107530-107538 |  |  |
| 190 | IGS *ccs*A - *ndh*D | (T)9 | 108151-108159 | (T)9 | 111364-111372 |
| 191 | IGS *psa*C - *ndh*E | (TTTA)3 | 110381-110392 |  |  |
| 192 | *psa*C | (TTGA)3 | 110443-110454 | (TTGA)3 | 113678-113689 |
| 193 | *ndh*G | (A)11 | 111125-111135 | (A)10 | 114348-114357 |
| 194 | IGS *ndh*G - *ndh*I | (A)8 | 111547-111554 | (A)8 | 114774-114781 |
| 195 | IGS *ndh*I - *ndh*A |  |  | (TTA)4 | 115384-115395 |
| 196 | *ndh*I | (T)12 | 111811-111822 |  |  |
| 197 | (intron) *ndh*A |  |  | (T)10 | 116752-116761 |
| 198 | *ndh*A | (T)11 | 113497-113507 |  |  |
| 199 | *ndh*A | (A)13 | 113582-113594 | (A)17 | 116833-116849 |
| 200 | *ndh*A |  |  | (ATTT)3 | 116875-116886 |
| 201 | IGS *rps*15 - *ycf*1 | (A)15 | 115603-115617 | (A)11 | 118859-118869 |
| 202 | *ycf*1 | (AT)4 | 116122-116129 | (AT)4 | 119364-119371 |
| 203 | *ycf*1 | (T)12 | 117092-117103 | (T)12 | 120331-120342 |
| 204 | *ycf*1 | (T)10 | 117237-117246 | (T)11 | 120476-120486 |
| 205 | *ycf*1 | (T)8 | 117653-117660 | (T)8 | 120883-120890 |
| 206 | *ycf*1 | (T)9 | 117879-117887 | (T)9 | 121109-121117 |
| 207 | *ycf*1 | (A)10 | 118286-118295 | (A)10 | 121528-121537 |
| 208 | *ycf*1 | (T)8 | 118349-118356 | (T)8 | 121591-121598 |
| 209 | *ycf*1 | (AT)4(T)12* | 118775-118793 |  |  |
| 210 | *ycf*1 |  |  | (T)10 | 122022-122031 |
| 211 | *ycf*1 | (T)8 | 118828-118835 | (T)8 | 122070-122077 |
| 212 | *ycf*1 | (T)12 | 119079-119090 | (T)11 | 122321-122331 |
| 213 | *ycf*1 | (T)8 | 119450-119457 | (T)8 | 122692-122699 |
| 214 | *ycf*1 | (A)9 | 119550-119558 | (A)9 | 122792-122800 |
| 215 | *ycf*1 | (T)15 | 120185-120199 | (T)15 | 123427-123441 |
| 216 | *ycf*1 | (T)9 | 120217-120225 | (T)10 | 123458-123467 |
| 217 | *ycf*1 | (T)10 | 120306-120315 | (T)10 | 123548-123557 |
| 218 | *ycf*1 | (A)12 | 120461-120472 | (A)12 | 123703-123714 |
| 219 | *ycf*1 | (A)9 | 120555-120563 | (A)9 | 123797-123805 |
| 220 | *rrn*23 | (AG)4 | 124442-124449 | (AG)4 | 127711-127718 |
| 221 | IGS *trn*V-GAC - *rps*12 | (T)8 | 130525-130532 | (T)8 | 133802-133809 |
| 222 | IGS *trn*V-GAC - *rps*12 | (A)12 | 130572-130583 | (A)11 | 133849-133859 |
| 223 | *ndh*B | (CT)4 | 135216-135223 | (CT)4 | 138530-138537 |
| 224 | IGS *ndh*B - *trn*L-CAA | (TA)5 | 135897-135906 | (TA)5 | 139261-139270 |
| 225 | *ycf*2 | (TC)5 | 140745-140754 | (TC)5 | 144165-144174 |
| 226 | *ycf*2 | (T)9 | 140767-140775 | (T)9 | 144187-144195 |
| 227 | *ycf*2 | (T)8 | 142009-142016 | (T)8 | 145429-145436 |
| 228 | *ycf*2 | (TC)4 | 142890-142897 | (TC)4 | 146310-146317 |
| 229 | IGS *trn*H-GUG – *rps*19 | (A)11 | 146219-146229 | (A)11 | 149630-149640 |
| 230 | IGS *trn*H-GUG - *rps*19 | (A)9 | 146257-146265 | (A)9 | 149668-149676 |
| 231 | *rps*19 | (TATTT)3 | 146604-146618 |  |  |
