# Supplementary material for: The complete plastid genomes of Ophrys iricolor and O. sphegodes (Orchidaceae) and comparative analyses with other orchids
Source: PLoS One. 2018 Sep 18;13(9):e0204174. doi: 10.1371/journal.pone.0204174 (PMC6143245; doi:10.1371/journal.pone.0204174)
Supplement: S2 Table — High dashes indicate absence of RNA editing, * stop codon. (DOCX) [file pone.0204174.s002.docx]

**S2 Table** List of RNA editing sites predicted in protein-coding genes of *Ophrys* plastomes using PREPACT program. High dashes indicate absence of RNA editing, * stop codon.

| **Gene** | **Nt pos.** | **AA pos.** | ***O. sphegodes O. iricolor*** | | **AA change** |
| --- | --- | --- | --- | --- | --- |
| *acc*D | 748  1184  1306  1370  1376  1412 | 250  395  436  57  459  471 | CAC→UAC  UCA→UUA  CAC→UAC  UCA→UUA  GCG→GUG  CCA→CUA | CAC→UAC  UCA→UUA  CAC→UAC  UCA→UUA  GCG→GUG  CCA→CUA | H→Y  S→L  H→Y  S→L  A→V  P→L |
| *atp*A | 773  914  1148  1493 | 258  305  383  498 | UCA→UUA  UCA→UUA  UCA→UUA  ACC→AUC | UCA→UUA  -  UCA→UUA  ACC→AUC | S→L  S→L  S→L  T→I |
| *atpB* | - | - | - | - | - |
| *atp*F | 92  248 | 31  83 | CCA→CUA  - | CCA→CUA  GCU→GUU | P→L  A→V |
| *atp*I | 428  437  629 | 143  146  210 | CCC→CUC  GCG→GUG  UCA→UUA | CCC→CUC  GCG→GUG  UCA→UUA | P→L  A→V  S→L |
| *ccs*A | 122  266  280  511  553 | 41  89  94  171  185 | UCA→UUA  CCG→CUG  CAU→UAU  -  - | UCA→UUA  CCG→CUG  CAU→UAU  CUU→UUU  CUU→UUU | S→L  P→L  H→Y  L→F  L→F |
| *clp*P | 82  263  559 | 28  88  187 | CAU→UAU  UCA→UUA  CAU→UAU | CAU→UAU  UCA→UUA  CAU→UAU | H→Y  S→L  H→Y |
| *mat*k | 331  472  656  722  872  913  916  953  1124  1186  1261  1460 | 111  158  219  241  291  305  306  318  375  396  421  487 | CCA→UCA  CAU→UAU  UCU→UUU  ACA→AUA  GCU→GUU  CAU→UAU  CUU→UUU  UCU→UUU  UCU→UUU  CCA→UCA  CAC→UAC  CCU→CUU | CCA→UCA  CAU→UAU  UCU→UUU  ACA→AUA  GCU→GUU  CAU→UAU  CUU→UUU  UCU→UUU  UCU→UUU  CCA→UCA  CAC→UAC  CCU→CUU | P→S  H→Y  S→F  T→I  A→V  H→Y  L→F  S→F  S→F  P→S  H→Y  P→L |
| *pet*B | 418  611 | 140  204 | CGG→UGG  CCA→CUA | CGG→UGG  CCA→CUA | R→W  P→L |
| *pet*D | 416 | 139 | GCA→GUA | GCA→GUA | A→V |
| *pet*G | - | - | - | - | - |
| *pet*L | 5 | 2 | CCU→CUU | CCU→CUU | P→L |
| *psa*B | 680  2132 | 227  711 | -  GCC→GUC | ACG→AUG  GCC→GUC | T→M  A→V |
| *psa*I | 80 | 27 | UCU→UUU | UCU→UUU | S→F |
| *psb*B | - | - | - | - | - |
| *psb*E | - | - | - | - | - |
| *psb*F | 77 | 26 | UCU→UUU | UCU→UUU | S→F |
| *psb*L | - | - | - | - | - |
| *rpl*2 | 2  31  217 | 1  11  73 | ACG→AUG  CCG→UCG  CCU→UCU | ACG→AUG  CCG→UCG  CCU→UCU | T→M  P→S  P→S |
| *rpl*20 | 241  287  352 | 81  96  118 | CUC→UUC  ACA→AUA  CAA→UAA | CUC→UUC  ACA→AUA  CAA→UAA | L→F  T→I  Q→* |
| *rpl*23 | 71 | 24 | UCU→UUU | UCU→UUU | S→F |
| *rpo*A | 200  368  778  830 | 67  123  260  277 | UCU→UUU  UCA→UUA  CUU→UUU  UCA→UUA | UCU→UUU  UCA→UUA  CUU→UUU  UCA→UUA | S→F  S→L  L→F  S→L |
| *rpo*B | 29  179  338  551  623  1736  1747  2426 | 10  60  113  184  208  579  583  809 | UCC→UUC  GCA→GUA  UCU→UUU  UCA→UUA  CCG→CUG  GCC→GUC  CGC→UGC  UCA→UUA | UCC→UUC  GCA→GUA  UCU→UUU  UCA→UUA  CCG→CUG  GCC→GUC  CGC→UGC  UCA→UUA | S→F  A→V  S→F  S→L  P→L  A→V  R→C  S→L |
| *rpo*C1 | 41  182  257  488  617  787  1622  1742  1948 | 14  61  86  163  206  263  541  581  650 | CCA→CUA  UCC→UUC  UCU→UUU  UCA→UUA  UCG→UUG  CGG→UGG  GCA→GUA  CCG→CUG  CGU→UGU | CCA→CUA  UCC→UUC  UCU→UUU  UCA→UUA  UCG→UUG  CGG→UGG  GCA→GUA  CCG→CUG  CGU→UGU | P→L  S→F  S→F  S→L  S→L  R→W  A→V  P→L  R→C |
| *rpo*C2 | 767  1628  1970  2078  2596  3011  3725  4016 | 256  543  657  693  866  1004  1242  1339 | CCA→CUA  ACC→AUC  ACG→AUG  GCU→GUU  CGU→UGU  UCA→UUA  UCA→UUA  ACU→AUU | CCA→CUA  ACC→AUC  ACG→AUG  GCU→GUU  CGU→UGU  UCA→UUA  UCA→UUA  ACU→AUU | P→L  T→I  T→M  A→V  R→C  S→L  S→L  T→I |
| *rps*2 | 134 | 45 | ACA→AUA | ACA→AUA | T→I |
| *rps*8 | 182 | 61 | UCA→UUA | UCA→UUA | S→L |
| *rps*14 | 80  149 | 27  50 | UCA→UUA  - | UCA→UUA  CCA→CUA | S→L  P→L |
| *rps*16 | 143  202 | 8  68 | UCA→UUA  CAU→UAU | UCA→UUA  CAU→UAU | S→L  H→Y |
| *ycf*3 | 44  185  191 | 15  62  64 | UCU→UUU  ACG→AUG  CCA→CUA | UCU→UUU  ACG→AUG  CCA→CUA | S→F  T→M  P→L |
