# Supplementary material for: The complete plastid genomes of Ophrys iricolor and O. sphegodes (Orchidaceae) and comparative analyses with other orchids
Source: PLoS One. 2018 Sep 18;13(9):e0204174. doi: 10.1371/journal.pone.0204174 (PMC6143245; doi:10.1371/journal.pone.0204174)
Supplement: S3 Table — “Null” and “positive” columns list likelihood values obtained under the models M8a (null model) and M8 (positive selection), respectively. (DOCX) [file pone.0204174.s003.docx]

S3 Table 3 Positive selection sites identified with selecton with d.f. = 1. “Null” and “positive” columns list likelihood values obtained under the models M8a (null model) and M8 (positive selection), respectively.

| Gene | Null | Positive | Putative sites under positive selection * |
| --- | --- | --- | --- |
| *acc*D | -9228,01 | -9205,28 | 95 (1 M, 2 E, 4 C, 5 W, 8 L, 9 M, 10 L, 11 S, 12 N, 13 K, 18 R, 20 G, 25 K, 30 A, 32 A, 36 T, 44 L, 47 A, 48 E, 50 K, 52 P, 54 W, 55 G, 56 S, 57 Y, 59 L, 63 H, 65 L, 67 S, 68 F, 71 S, 75 W, 86 R, 95 V, 100 E, 102 Q, 113 L, 121 F, 122 N, 124 N, 126 S, 127 G, 129 L, 142 R, 145 P, 149 F, 152 T, 155 R, 159 E, 167 Y, 169 G, 170 I, 171 E, 172 N, 173 Y, 175 T, 180 A, 183 I, 188 D, 189 E, 191 L, 194 S, 196 S, 197 F, 199 R, 200 R, 201 E, 202 I, 206 F, 208 I, 221 E, 222 T, 229 R, 230 S, 237 H, 253 F, 257 G, 269 M, 282 R, 286 Y, 290 I, 302 I, 304 R, 309 P, 318 Q, 374 S, 379 S, 380 N, 386 V, 436 S, 438 A, 442 L, 456 L, 475 Q, 476 G) |
| *atp*I | -1868,92 | -1864,95 | 12 (8 I, 9 K, 26 L, 29 Q, 54 V, 63 T, 67 D, 80 R, 143 P, 146 A, 154 S, 162 G) |
| *ccs*A | -4187,25 | -4177,65 | 37 (4 V, 5 T, 23 M, 32 Y, 38 R, 42 E, 50 L, 87 R, 89 P, 91 L, 92 G, 94 H, 106 A, 113 A, 125 A, 134 Q, 149 A, 166 R, 168 N, 170 N, 171 L, 173 L, 175 N, 176 K, 179 F, 184 F, 193 F, 197 G, 199 V, 202 K, 206 L, 209 Q, 219 R, 280 S, 282 L, 286 G, 298 I) |
| *mat*K | -5982,85 | -5970,71 | 74 (6 L, 11 V, 13 Y, 16 Q, 18 I, 20 W, 21 G, 25 Y, 29 H, 31 Y, 35 L, 49 S, 50 Q, 52 V, 54 S, 57 E, 63 S, 64 Q, 81 S, 97 M, 119 L, 120 I, 127 L, 130 V, 136 D, 139 V, 143 R, 144 N, 146 R, 149 W, 159 C, 162 I, 164 L, 169 Q, 186 H, 189 V, 193 Y, 195 R, 203 Q, 204 R, 209 L, 212 P, 223 A, 226 A, 235 N, 238 K, 241 L, 245 W, 264 P, 271 M, 278 L, 281 T, 298 M, 299 T, 300 K, 306 V, 319 Q, 324 L, 342 L, 344 R, 363 Q, 398 G, 402 E, 405 M, 410 A, 413 L, 419 R, 420 P, 422 P, 425 G, 426 L, 438 R, 439 I, 445 H) |
| *pet*B | -1772,77 | -1759,86 | 6 (1 I, 2 N, 123 I, 140 R, 163 S, 204 P) |
| *psb*A | -2650,24 | -2640,4 | 1 (346 V) |
| *psb*B | -4099,82 | -4085,44 | 5 (296 L, 345 F, 352 R, 494 T, 504 R) |
| *psb*E | -570,09 | -565,51 | 2 (59 S, 78 E) |
| *psb*H | -660,86 | -651,56 | 5 (5 T, 16 R, 18 G, 45 V, 72 M) |
| *psb*M | -246,99 | -243,32 | 9 (6 L, 8 L, 12 A, 13 L, 24 I, 26 Y, 30 A, 33 N, 34 N) |
| *psb*N | -275,24 | -270,24 | 1 (32 Q) |
| *rbc*L | -3913,24 | -3891,32 | 14 (89 A, 142 P, 219 L, 225 L, 226 Y, 251 M, 375 L, 443 K, 449 S, 461 I, 470 D, 471 P, 475 L, 477 K) |
| *rpl*20 | -1609,01 | -1603,49 | 7 (75 F, 80 K, 81 L, 83 H, 112 F, 116 I, 118 Q) |
| *rpl*22 | -1487,53 | -1479,43 | 16 (7 S, 8 E, 10 S, 22 R, 26 F, 53 R, 71 N, 91 A, 93 M, 96 L, 98 P, 106 M, 110 T, 120 E, 122 S, 124 I) |
| *rpl*32 | -825,55 | -819,47 | 8 (19 L, 40 T, 42 Q, 49 R, 53 V, 54 L, 55 E, 57 S) |
| *rpl*33 | -866,07 | -861,89 | 9 (20 R, 23 V, 26 E, 27 S, 28 T, 45 R, 48 L, 49 K, 53 R) |
| *rpo*A | -3546,32 | -3534,9 | 42 (9 S, 14 Q, 25 K, 33 V, 34 M, 61 C, 64 C, 66 T, 71 L, 97 R, 105 D, 115 Y, 145 L, 146 C, 152 N, 154 D, 158 R, 164 N, 166 H, 167 D, 182 V, 190 G, 201 L, 237 M, 239 F, 240 E, 246 W, 250 P, 256 R, 260 L, 261 K, 266 G, 282 R, 283 T, 301 Y, 307 M, 309 M, 310 E, 311 Y, 313 C, 319 H, 322 S) |
| *rpo*B | -8918,47 | -8910,72 | 56 (2 L, 5 V, 24 C, 32 A, 36 Q, 54 V, 58 Q, 63 L, 85 V, 101 L, 158 L, 189 L, 212 E, 250 K, 268 R, 275 S, 325 F, 335 A, 348 L, 380 R, 451 E, 459 E, 463 E, 467 V, 468 F, 489 R, 581 A, 583 R, 589 Y, 596 V, 597 F, 604 L, 613 R, 625 Q, 627 R, 632 I, 638 I, 640 G, 699 S, 708 E, 746 T, 748 N, 753 A, 775 L, 796 G, 799 Y, 801 S, 803 R, 879 N, 910 Q, 935 L, 938 Q, 1020 L, 1023 M, 1027 S, 1065 I) |
| *rpo*C1 | -6089,39 | -6079,39 | 31 (21 R, 61 S, 76 V, 79 T, 83 D, 84 P, 129 L, 139 G, 148 N, 150 S, 154 S, 156 V, 210 S, 231 S, 259 I, 267 R, 423 V, 432 S, 446 Q, 548 M, 564 Y, 569 F, 573 T, 575 D, 585 P, 603 N, 608 L, 636 Y, 642 H, 644 Q, 680 R) |
| *rpo*C2 | -14495,1 | -14479,8 | 153 (8 V, 19 M, 26 L, 42 V, 44 T, 49 R, 223 I, 231 L, 233 G, 238 I, 256 P, 278 R, 379 L, 384 L, 386 I, 394 L, 424 A, 427 S, 434 R, 436 R, 453 G, 457 P, 480 L, 481 C, 486 V, 497 M, 504 F, 512 L, 533 K, 535 I, 536 D, 542 R, 543 T, 545 S, 551 L, 554 P, 557 F, 561 D, 563 Y, 566 S, 568 A, 587 N, 589 L, 592 C, 601 R, 622 S, 632 V, 637 M, 648 G, 649 T, 659 Q, 664 Q, 680 P, 699 L, 712 E, 718 M, 730 P, 732 E, 733 M, 738 R, 747 G, 749 E, 753 S, 759 F, 769 A, 771 T, 775 Y, 783 I, 798 S, 799 Q, 821 G, 848 R, 882 S, 890 T, 891 A, 895 L, 897 S, 899 S, 900 E, 904 I, 905 H, 906 I, 913 V, 916 Q, 917 S, 919 P, 922 R, 924 G, 926 F, 932 R, 936 C, 937 K, 940 I, 948 F, 950 T, 951 G, 952 P, 964 E, 965 A, 968 I, 969 I, 970 S, 974 L, 977 P, 986 V, 988 F, 989 C, 992 Y, 1000 V, 1003 K, 1006 L, 1007 S, 1017 V, 1022 T, 1024 K, 1032 R, 1033 R, 1035 Y, 1040 C, 1042 K, 1045 W, 1047 L, 1049 H, 1058 D, 1059 Y, 1060 Y, 1063 G, 1064 W, 1068 N, 1080 L, 1107 P, 1138 S, 1174 D, 1179 K, 1220 R, 1245 S, 1249 L, 1330 K, 1344 L, 1346 I, 1348 K, 1349 K, 1351 I, 1357 R, 1362 H, 1366 L, 1368 C, 1371 G, 1373 K, 1375 F, 1377 E, 1379 S, 1380 N) |
| *rps*12 | -850,70 | -830,32 | 8 (13 Q, 16 K, 18 I, 57 L, 88 K, 116 S, 117 A, 118 L) |
| *rps*19 | -682,64 | -679,31 | 10 (16 S, 17 E, 26 E, 27 E, 65 R, 78 L, 81 V, 82 R, 84 A, 88 N) |
| *ycf*1 | -29977,7 | -29875,2 | 505 (3 F, 7 L, 27 L, 32 L, 43 F, 48 R, 54 S, 66 A, 102 F, 104 W, 107 H, 117 T, 139 F, 148 T, 180 L, 193 R, 196 H, 200 S, 236 V, 243 T, 253 S, 259 Y, 271 S, 277 S, 283 E, 284 E, 292 H, 294 K, 295 E, 297 R, 307 S, 309 L, 311 T, 314 E, 316 W, 317 K, 318 L, 319 G, 321 P, 326 R, 327 I, 328 N, 329 I, 330 N, 331 K, 332 K, 333 I, 334 D, 335 I, 336 I, 337 Y, 338 L, 339 W, 340 V, 342 K, 345 I, 348 F, 353 R, 364 D, 383 K, 389 K, 402 S, 405 L, 407 R, 408 K, 410 S, 416 K, 418 L, 419 L, 427 T, 433 C, 435 L, 441 S, 444 Q, 446 L, 451 R, 453 P, 455 L, 462 N, 473 C, 474 L, 477 A, 482 L, 485 P, 489 T, 490 I, 493 L, 496 R, 497 T, 501 T, 503 T, 507 D, 508 L, 513 L, 528 L, 529 C, 530 R, 532 S, 534 L, 536 S, 542 S, 544 N, 545 K, 546 E, 548 Y, 549 L, 552 L, 553 F, 558 T, 559 H, 562 D, 563 Q, 565 I, 566 M, 568 K, 569 K, 570 S, 572 V, 574 K, 575 R, 577 E, 578 V, 584 Q, 591 E, 593 F, 596 E, 600 F, 601 T, 605 S, 606 G, 608 N, 612 A, 614 R, 615 T, 616 I, 619 E, 621 A, 622 N, 623 P, 628 T, 631 I, 632 T, 635 N, 637 S, 640 F, 661 C, 662 N, 663 L, 668 L, 671 P, 680 T, 681 D, 684 L, 685 F, 686 F, 691 K, 694 L, 695 L, 696 F, 699 W, 700 M, 701 G, 702 I, 706 D, 712 K, 714 E, 715 E, 717 K, 718 D, 720 N, 722 E, 724 E, 726 S, 728 I, 731 A, 733 L, 735 T, 737 A, 740 S, 741 F, 742 T, 744 L, 745 I, 749 L, 754 I, 757 L, 761 A, 765 L, 770 L, 772 I, 775 W, 776 H, 779 F, 786 K, 791 T, 800 T, 803 P, 804 Q, 808 T, 809 D, 816 I, 817 H, 827 S, 829 V, 830 R, 832 H, 833 H, 834 I, 836 Q, 837 M, 841 K, 844 Q, 845 N, 854 T, 856 T, 857 K, 858 I, 859 P, 862 S, 863 L, 866 K, 868 L, 871 K, 877 L, 878 K, 880 I, 884 V, 886 N, 887 K, 889 F, 890 Q, 892 I, 894 F, 895 L, 898 K, 899 R, 901 L, 904 K, 908 I, 910 W, 911 V, 912 I, 915 I, 916 R, 922 I, 925 I, 928 V, 930 L, 932 L, 933 F, 936 L, 944 P, 945 N, 948 N, 951 L, 955 N, 962 P, 968 M, 970 W, 971 L, 973 Y, 978 R, 980 I, 988 I, 991 K, 993 Q, 996 Q, 997 T, 998 E, 1000 E, 1007 Y, 1011 I, 1012 L, 1013 K, 1015 Y, 1017 H, 1018 L, 1019 W, 1024 R, 1034 H, 1037 I, 1050 V, 1052 S, 1054 T, 1055 F, 1056 F, 1058 I, 1060 A, 1063 L, 1069 N, 1072 N, 1074 S, 1076 Y, 1079 K, 1080 R, 1082 Q, 1083 K, 1084 K, 1086 G, 1088 N, 1090 I, 1092 Q, 1094 K, 1097 L, 1098 I, 1099 L, 1104 F, 1106 T, 1112 E, 1114 R, 1116 Q, 1118 S, 1120 I, 1121 Y, 1122 W, 1126 S, 1141 L, 1142 F, 1145 Y, 1147 L, 1149 P, 1156 N, 1167 Y, 1168 C, 1172 G, 1176 P, 1178 S, 1181 K, 1184 H, 1197 S, 1203 I, 1205 Q, 1209 R, 1211 I, 1215 W, 1216 R, 1219 R, 1221 K, 1222 L, 1223 R, 1233 L, 1239 F, 1240 N, 1241 S, 1246 D, 1249 A, 1257 D, 1259 C, 1274 N, 1275 P, 1276 P, 1278 S, 1281 S, 1282 E, 1288 K, 1289 E, 1290 A, 1291 K, 1296 H, 1297 F, 1299 T, 1300 S, 1317 L, 1320 K, 1322 I, 1325 S, 1329 L, 1336 C, 1340 S, 1341 I, 1342 C, 1344 R, 1348 E, 1350 W, 1351 T, 1353 A, 1356 R, 1357 R, 1358 N, 1360 Y, 1365 T, 1368 H, 1370 N, 1374 M, 1376 H, 1377 Q, 1378 K, 1381 P, 1382 C, 1386 R, 1387 N, 1396 K, 1398 R, 1403 E, 1406 H, 1407 A, 1411 T, 1418 G, 1421 F, 1423 V, 1425 K, 1427 K, 1430 I, 1438 L, 1439 N, 1441 D, 1442 A, 1443 N, 1444 E, 1451 R, 1457 L, 1459 V, 1460 G, 1462 F, 1463 E, 1467 H, 1468 E, 1470 Q, 1471 N, 1473 G, 1476 V, 1477 L, 1480 L, 1483 Q, 1484 N, 1486 K, 1487 A, 1493 R, 1494 K, 1495 F, 1497 M, 1500 S, 1501 K, 1507 T, 1511 M, 1515 S, 1519 N, 1520 S, 1527 W, 1528 I, 1529 N, 1530 F, 1531 S, 1533 E, 1534 K, 1540 R, 1541 T, 1548 V, 1549 K, 1551 I, 1555 A, 1558 S, 1560 K, 1562 D, 1566 L, 1570 F, 1573 K, 1574 D, 1576 V, 1578 K, 1586 F, 1587 L, 1592 C, 1593 L, 1596 R, 1598 D, 1599 G, 1601 S, 1612 V, 1613 D, 1616 H, 1620 N, 1623 T, 1629 E, 1631 G, 1632 E, 1633 L, 1634 K, 1636 Y, 1638 V, 1639 R, 1640 H, 1642 N, 1645 F, 1647 G, 1651 N, 1654 F, 1656 I, 1672 R, 1675 L, 1677 S, 1678 K, 1680 C, 1683 A, 1687 P, 1690 C, 1702 L, 1704 E, 1705 D, 1712 E, 1714 N, 1715 L, 1716 M, 1718 L, 1744 S) |
| *ycf*2 | -18981,9 | -18817,4 | 654 (3 R, 6 F, 7 K, 8 S, 11 F, 13 F, 22 L, 28 K, 30 N, 39 F, 46 M, 54 W, 55 S, 63 R, 66 T, 67 S, 72 T, 74 K, 76 V, 77 V, 80 V, 81 V, 82 V, 84 L, 85 I, 86 S, 92 K, 99 L, 103 G, 108 P, 115 I, 124 W, 125 S, 128 R, 139 P, 141 G, 143 K, 144 I, 145 S, 146 D, 148 C, 150 M, 157 W, 158 V, 159 L, 161 I, 163 Q, 165 C, 175 R, 181 N, 182 R, 183 Y, 184 F, 185 G, 186 K, 187 T, 190 Q, 192 L, 196 V, 208 S, 214 L, 217 S, 229 W, 231 F, 243 I, 253 E, 258 D, 259 L, 261 C, 266 A, 271 R, 274 H, 275 F, 276 L, 279 Q, 302 W, 309 C, 310 A, 311 Q, 330 Y, 335 L, 345 W, 362 G, 369 Q, 373 T, 374 R, 379 Q, 387 K, 389 S, 390 Y, 400 S, 402 R, 404 E, 416 E, 418 Q, 420 L, 431 F, 434 T, 438 E, 448 L, 451 S, 458 F, 462 E, 466 N, 475 E, 476 E, 487 Y, 498 L, 502 P, 506 S, 507 T, 508 I, 510 Q, 512 L, 514 K, 515 K, 519 V, 522 V, 523 P, 527 V, 529 N, 530 Q, 536 F, 544 N, 546 V, 554 D, 555 P, 556 G, 557 C, 559 M, 564 E, 571 N, 576 L, 577 N, 580 P, 581 F, 582 F, 583 D, 584 F, 585 F, 586 H, 588 F, 589 H, 590 D, 591 R, 592 N, 593 K, 594 G, 595 G, 596 Y, 597 A, 598 L, 599 R, 600 H, 603 F, 624 Y, 625 H, 628 S, 634 K, 635 K, 636 F, 647 S, 649 N, 652 L, 667 S, 671 I, 673 K, 674 S, 675 V, 690 T, 691 A, 692 V, 702 V, 704 Q, 723 R, 728 R, 731 L, 740 E, 745 R, 758 I, 762 T, 765 R, 767 L, 770 F, 772 N, 773 S, 780 P, 783 S, 784 R, 785 T, 787 R, 791 W, 795 A, 800 W, 803 G, 804 S, 809 E, 818 P, 820 Q, 824 A, 828 R, 830 R, 831 I, 833 Q, 835 S, 841 A, 845 E, 846 D, 847 L, 848 S, 850 S, 854 F, 858 S, 860 P, 864 V, 873 R, 877 H, 878 I, 881 L, 884 P, 889 C, 891 Q, 895 S, 905 K, 909 F, 910 L, 917 S, 922 F, 926 G, 930 L, 933 L, 937 I, 941 M, 943 D, 952 G, 954 S, 958 T, 961 Y, 962 F, 965 I, 972 W, 978 P, 985 I, 987 S, 989 Y, 1002 H, 1010 R, 1014 D, 1021 N, 1044 C, 1045 A, 1049 K, 1050 D, 1051 L, 1057 T, 1061 I, 1072 N, 1074 F, 1081 T, 1084 L, 1088 L, 1091 P, 1093 G, 1096 P, 1100 R, 1106 A, 1110 A, 1111 T, 1114 T, 1118 I, 1119 V, 1123 R, 1125 Y, 1128 P, 1139 R, 1140 N, 1145 Y, 1151 N, 1157 T, 1158 P, 1161 E, 1163 Y, 1165 P, 1166 S, 1175 C, 1177 K, 1184 Q, 1186 Y, 1188 T, 1189 F, 1190 Q, 1198 L, 1206 T, 1211 F, 1227 T, 1230 D, 1231 P, 1234 I, 1237 S, 1240 K, 1254 I, 1255 L, 1256 R, 1257 P, 1259 T, 1261 K, 1264 T, 1266 W, 1267 T, 1268 L, 1271 E, 1276 C, 1277 L, 1278 Q, 1281 L, 1282 L, 1283 S, 1284 E, 1286 M, 1290 K, 1295 I, 1297 L, 1299 W, 1300 A, 1303 R, 1307 A, 1312 Y, 1316 F, 1320 V, 1324 L, 1325 V, 1326 R, 1330 L, 1332 V, 1334 R, 1335 A, 1336 S, 1338 E, 1345 K, 1348 S, 1350 M, 1352 P, 1354 Y, 1356 M, 1358 F, 1359 R, 1360 K, 1361 L, 1362 L, 1370 L, 1372 S, 1375 L, 1381 V, 1382 V, 1383 L, 1384 E, 1385 Q, 1387 G, 1389 S, 1391 E, 1392 E, 1395 G, 1396 S, 1397 A, 1398 S, 1399 G, 1400 G, 1404 W, 1405 G, 1406 G, 1407 A, 1409 G, 1410 V, 1413 I, 1415 S, 1417 K, 1418 K, 1420 W, 1421 K, 1431 I, 1437 R, 1439 I, 1442 R, 1451 S, 1454 I, 1459 R, 1461 R, 1464 V, 1465 N, 1466 G, 1467 D, 1468 W, 1471 E, 1475 F, 1476 W, 1477 V, 1479 N, 1481 D, 1482 S, 1484 D, 1485 D, 1486 E, 1488 R, 1489 E, 1490 F, 1492 V, 1496 T, 1502 R, 1505 K, 1506 I, 1514 D, 1517 S, 1518 K, 1529 P, 1533 S, 1537 L, 1542 K, 1554 C, 1566 Q, 1569 A, 1570 Y, 1574 S, 1575 C, 1576 G, 1577 A, 1578 N, 1585 P, 1590 R, 1592 A, 1593 L, 1594 S, 1595 P, 1607 T, 1615 Y, 1620 S, 1622 V, 1629 P, 1630 N, 1632 F, 1633 L, 1639 G, 1640 Y, 1641 P, 1642 I, 1645 S, 1646 D, 1647 D, 1648 T, 1650 I, 1652 D, 1653 S, 1655 D, 1656 T, 1658 I, 1659 D, 1661 S, 1662 D, 1664 I, 1665 Y, 1668 G, 1669 S, 1670 D, 1671 D, 1672 D, 1673 L, 1677 T, 1678 E, 1679 L, 1680 L, 1681 T, 1684 M, 1685 T, 1686 P, 1687 N, 1688 I, 1689 D, 1690 Q, 1691 F, 1692 D, 1693 I, 1694 T, 1695 L, 1699 L, 1702 A, 1708 I, 1717 H, 1718 V, 1720 E, 1723 Y, 1724 L, 1725 S, 1726 L, 1727 G, 1730 E, 1737 C, 1741 S, 1755 Q, 1756 K, 1760 A, 1765 K, 1767 L, 1773 I, 1775 K, 1777 L, 1778 L, 1782 R, 1787 T, 1799 R, 1803 T, 1807 G, 1808 S, 1809 I, 1815 A, 1816 R, 1819 V, 1820 A, 1821 L, 1824 E, 1825 A, 1831 T, 1833 K, 1835 Y, 1839 T, 1845 A, 1846 L, 1848 R, 1849 K, 1852 D, 1855 S, 1856 Q, 1860 V, 1862 D, 1872 R, 1874 V, 1875 A, 1879 L, 1883 C, 1893 K, 1894 K, 1895 N, 1897 C, 1898 K, 1899 G, 1902 S, 1903 D, 1912 G, 1913 T, 1914 S, 1917 K, 1918 F, 1942 P, 1946 N, 1947 W, 1949 T, 1954 V, 1958 S, 1967 L, 1970 L, 1972 V, 1974 G, 1976 P, 1978 L, 1979 A, 1980 G, 1982 S, 1987 D, 1990 Q, 2000 L, 2002 S, 2007 Q, 2012 Q, 2015 S, 2018 T, 2019 V, 2021 Q, 2022 R, 2024 L, 2027 K, 2028 Y, 2029 E, 2030 S, 2031 E, 2036 A, 2037 L, 2039 P, 2040 Q, 2041 Q, 2042 I, 2044 E, 2045 D, 2046 L, 2049 H, 2055 R, 2063 E, 2065 P, 2071 P, 2073 W, 2074 I, 2078 R, 2081 R, 2082 I, 2084 S, 2089 E, 2091 Q, 2104 Q, 2106 Q, 2107 T, 2108 R, 2111 S, 2112 S, 2113 K, 2114 E, 2115 Q, 2116 G, 2117 F, 2118 F, 2119 R, 2120 T, 2121 S, 2125 W, 2128 A, 2130 P, 2131 L, 2135 F, 2136 K, 2138 Q, 2139 P, 2140 F, 2141 V, 2143 V, 2156 S, 2158 G, 2160 I, 2163 Q, 2164 T, 2166 P, 2167 P, 2170 M, 2173 R, 2180 Q, 2191 Q, 2192 R, 2194 F, 2203 G, 2209 T, 2211 S, 2220 L, 2225 G, 2234 T, 2239 R, 2241 L, 2243 P, 2254 G, 2257 F) |

* Lower bound > 1
