# Supplementary figures and images for: The complete plastid genomes of Ophrys iricolor and O. sphegodes (Orchidaceae) and comparative analyses with other orchids
Source: PLoS One. 2018 Sep 18;13(9):e0204174. doi: 10.1371/journal.pone.0204174 (PMC6143245; doi:10.1371/journal.pone.0204174)

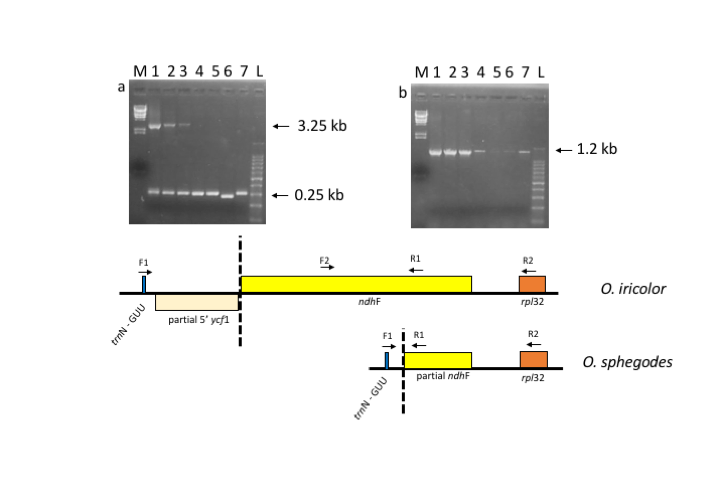

Supplement: S1 Fig — PCR amplifications using (a) F1 and R1 primers; (b) F2 and R2 primers. M = marker II (λ DNA / Hind III digested); 1 = O. fusca Campania, 2 = O. fusca Tuscany, 3 = O. iricolor Greece; 4 = O. sphegodes Campania; 5 = O. sphegodes Apulia; 6 = O. incubacea Apulia; 7 = O. insectifera Spain. A dotted line represents the IRB-SSC junction. Primer sequences: F1: 5’—GCTCCGTTCCATGCCTCATT– 3’ R1: 5’–TCGTCGTATGTGGGCTTTCC– 3’ F2: 5’–TTAGCAATTGCACCGACAAA– 3’ R2: 5’–TCTGTTTCCACCGGACAG– 3’. (TIFF) [file pone.0204174.s004.tiff]

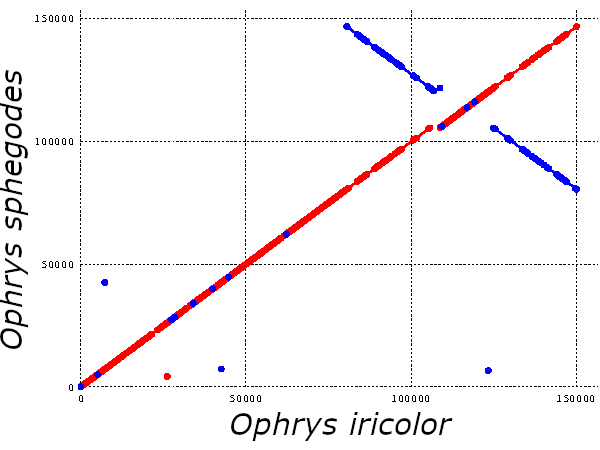

Supplement: S4 Fig — A positive slope indicates that compared sequences are in the same orientation; a negative slope indicates that compared sequences can be aligned, but their orientation is opposite. Red: Sequences in the same direction; Blue: inversions. (JPG) [file pone.0204174.s007.jpg]
